# Identification of conserved drought-adaptive genes using a cross-species meta-analysis approach

**DOI:** 10.1186/s12870-015-0493-6

**Published:** 2015-05-03

**Authors:** Lidor Shaar-Moshe, Sariel Hübner, Zvi Peleg

**Affiliations:** The Robert H. Smith Institute of Plant Sciences and Genetics in Agriculture, The Hebrew University of Jerusalem, Rehovot, 7610001 Israel; Present address: Department of Botany, University of British Columbia, Vancouver, BC Canada

**Keywords:** *Brachypodium distachyon*, Cross-species meta-analysis, Drought stress, Evolutionary conservation, Microarray, Osmotic adjustment

## Abstract

**Background:**

Drought is the major environmental stress threatening crop-plant productivity worldwide. Identification of new genes and metabolic pathways involved in plant adaptation to progressive drought stress at the reproductive stage is of great interest for agricultural research.

**Results:**

We developed a novel **C**ross-**S**pecies meta-**A**nalysis of progressive **Drought** stress at the reproductive stage (**CSA:Drought**) to identify key drought adaptive genes and mechanisms and to test their evolutionary conservation. Empirically defined filtering criteria were used to facilitate a robust integration of 17 deposited microarray experiments (148 arrays) of Arabidopsis, rice, wheat and barley. By prioritizing consistency over intensity, our approach was able to identify 225 differentially expressed genes shared across studies and taxa. Gene ontology enrichment and pathway analyses classified the shared genes into functional categories involved predominantly in metabolic processes (e.g. amino acid and carbohydrate metabolism), regulatory function (e.g. protein degradation and transcription) and response to stimulus. We further investigated drought related *cis*-acting elements in the shared gene promoters, and the evolutionary conservation of shared genes. The universal nature of the identified drought-adaptive genes was further validated in a fifth species, *Brachypodium distachyon* that was not included in the meta-analysis. qPCR analysis of 27, randomly selected, shared orthologs showed similar expression pattern as was found by the CSA:Drought.In accordance, morpho-physiological characterization of progressive drought stress, in *B. distachyon*, highlighted the key role of osmotic adjustment as evolutionary conserved drought-adaptive mechanism.

**Conclusions:**

Our CSA:Drought strategy highlights major drought-adaptive genes and metabolic pathways that were only partially, if at all, reported in the original studies included in the meta-analysis. These genes include a group of unclassified genes that could be involved in novel drought adaptation mechanisms. The identified shared genes can provide a useful resource for subsequent research to better understand the mechanisms involved in drought adaptation across-species and can serve as a potential set of molecular biomarkers for progressive drought experiments.

**Electronic supplementary material:**

The online version of this article (doi:10.1186/s12870-015-0493-6) contains supplementary material, which is available to authorized users.

## Background

Drought stress adversely affects plant growth and productivity worldwide. It is estimated that about 40% of all croplands are affected by moderate to extreme water stress (http://www.wri.org/applications/maps/agriculturemap). Moreover, agro-ecological conditions expected to deteriorate, due to foreseen global climatic changes, towards reduced availability and increased variability of water resources. The ever-increasing human population that is expected to exceed 9 billion people by 2050 (http://www.fao.org/wsfs/world-summit/en) together with the loss of agricultural land, poses serious challenges to agricultural plant research. Thus, developing drought-resistance crop-plants with enhanced productivity and improved water-use efficiency is the most promising solution for alleviating future threats to food security.

Plants have evolved various adaptive mechanisms to cope with drought stress at multiple levels such as molecular, cellular, tissue, anatomical, morphological and whole-plant physiological level [1-3]. Transcriptional profiling analyses, in various species, have been widely used to identify drought-related genes (e.g. [4-7]). These experiments resulted in condition- and/or genotype-specific genes with little overlaps across studies (reviewed by [8]).

Meta-analysis is a powerful strategy to exploit the potential of transcriptome studies [9]. The combination of multiple studies, addressing similar experimental setups, enhances the reliability of the results by increasing the statistical power to reveal a more valid and precise set of differentially expressed genes (DEGs) [10]. Moreover, combining gene expression information across species can improve the ability to identify core gene sets with high evolutionary conservation. These genes are conserved in both sequence and expression across multiple species and are thus key components of the biological responses being studied [11]. In animals, microarray meta-analyses have been extensively used for gene discovery (reviewed by [12,13]). However, only few microarray meta-analyses were reported in plants, with the majority conducted in Arabidopsis (*Arabidopsis thaliana*) [14-22]. Even fewer studies involved more than one plant species (e.g. [23-25]). To date, an extensive amount of transcriptome data, from various plant species, developmental stages, tissues and experimental conditions, are publicly available. Thus, re-analyzing published data using a meta-analysis and a cross-species approach could promote detection of conserved key genes and pathways that were overlooked using other analytical approaches and facilitate prediction of functional drought responses in non-model species.

In the current study, we developed a novel **C**ross-**S**pecies meta-**A**nalysis of progressive **Drought** stress at the reproductive stage (CSA:Drought), using Arabidopsis, rice, wheat and barley microarray studies. Based on this dataset we identified shared key genes and metabolic pathways involved in whole plant adaptation to progressive drought stress across-species. We further evaluated the level of sequence conservation between shared and species-specific DEGs and detected common regulatory *cis*-acting elements in their promoters. Finally, based on transcriptional and morpho-physiological analyses, we validated the universal nature and functional conservation of selected shared DEGs in a fifth species, *Brachypodium distachyon*.

## Results

### Meta-analysis of microarray progressive drought stress studies

**Figure 1:**
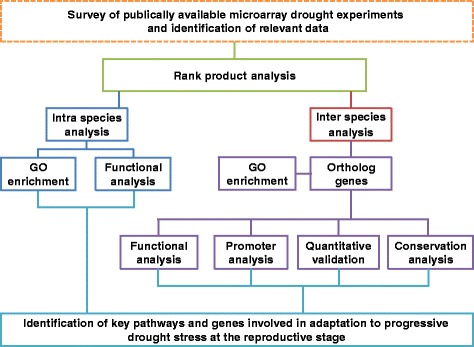
A schematic overview of the **C**ross-**S**pecies meta-**A**nalysis of progressive **Drought** stress at the reproductive stage (CSA:Drought) approach. Following selection of relevant microarray drought stress studies, raw data, from each species, was integrated into separate datasets using rank product analysis. This statistical method generated lists of up- and down-regulated genes based on their expression (i.e. rank) across the individual experiments within each species. Significantly differentially expressed genes (DEGs), were used for intra-species analysis to retrieve enriched gene ontology (GO) terms and to classify genes into functional pathways. Next, DEGs within each species were transformed to rice orthologs and the penalized Fisher method was used to combine *P*-value distributions across species meta-analysis. Finally, the shared drought-adaptive DEGs were characterized and their universal nature was validated in a fifth species that was not included in the meta-analysis.

A schematic workflow, summarizing each step of the CSA:Drought strategy is described in Figure 1. A wide survey of deposited drought related microarray studies, in various plant and crop species, was conducted. Focus was given to studies involving progressive drought stress at the reproductive stage. Most of the microarray studies found in databases (~4,000) were conducted in Arabidopsis (~3000), with only 15 studies involving drought stress at the reproductive stage. Among other plant species, only rice (10 studies), wheat (5 studies) and barley (2 studies) included more than one drought stress experiment at the reproductive stage. Altogether, 32 studies, conducted at the reproductive stage, from four different plant species, were found in our survey. To further homogenize the experimental setup, only Affymetrix GeneChip platform and aboveground tissues of soil grown wild type (WT) plants were included. It is worth noted that all selected Arabidopsis experiments used Col-0 ecotype, while, for other plants, different genotypes were included, due to low number of studies from the same genetic background (Additional file 1: Table S1). Following a hierarchical clustering analysis to assess the quality of the studies, additional eight arrays were removed due to inconsistent expression profile across biological replicates within the same experiment (Additional file 2: Figure S1). In total, 148 arrays corresponding to 17 progressive drought stress studies, from four different plant species, were included in the CSA:Drought pipeline (Table 1).

**Table 1:** **Overall summary of within species microarray meta-analysis**

**Plant species**	**Clade**	**Studies** ^**a**^	**Arrays**	**Probe-sets** ^**b**^	**DEGs** ^**c**^	**GOs** ^**d**^
**Arabidopsis**	Eudicot	8	40	22 k	3.5 k	663
**Rice**	Monocot	3	34	57 k	7.3 k	180
**Wheat**	Monocot	4	38	61 k	2.4 k	86
**Barley**	Monocot	2	36	22 k	2.7 k	27

^**a.**^ Details of the individual microarray studies that were included in the CSA:Drought is given in Additional file 1: Table S1.

^**b.**^ Affymetrix Genechip® Microarray of Arabidopsis, rice, wheat and barley.

^**c.**^ Differentially expressed genes, false-positive prediction (PFP) ≤ 0.05.

^**d.**^ Enriched gene ontology biological processes (FDR ≤ 0.05).

**Figure 2:**
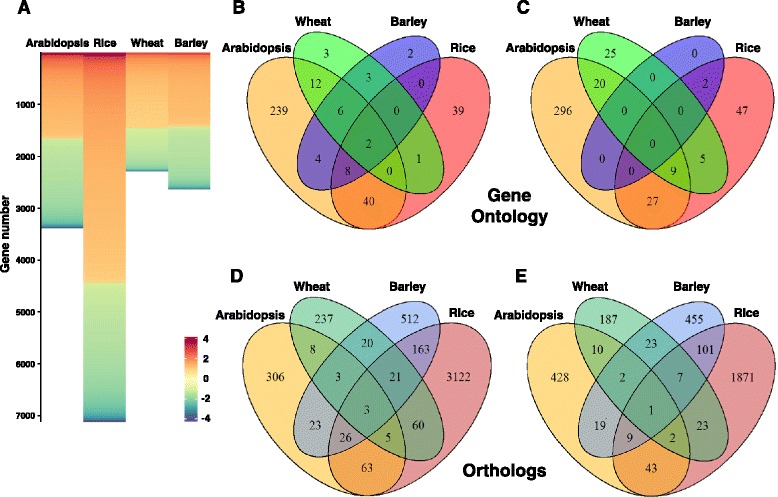
Within species microarray meta-analysis. **(A)** Expression profiles of significantly differentially expressed genes in each species based on the rank product analysis. Length of heatmap is proportional to number of probe-sets. Unique and common **(B)** up- and **(C)** down-regulated gene ontology biological processes (FDR ≤ 0.05) based on significantly differentially expressed genes within each species. Unique and common **(D)** up- and **(E)** down-regulated orthologs (FDR ≤ 0.05).

Microarray data from each species was integrated into a comparable meta-analysis platform using the rank product approach. The number of significant DEGs detected for Arabidopsis (3.5 k), rice (7.3 k), wheat (2.4 k) and barley (2.7 k) (Figure 2A and Additional file 3: Table S2) was not affected by the array size (r = −0.05, *P* = 0.9). However, the number of studies integrated in the meta-analysis affected the number of significant DEGs detected in each species (r = −0.88, *P* = 0.004). This effect is inherent to meta-analysis and was previously reported (e.g. [20]). Despite the negative effect of less overlapping DEGs when increasing number of studies, the improved statistical power and augmented stringency further supported the inclusion of more studies over the cost of false negative calls. The percentage of DEGs (with respect to the transcriptome size) highlighted Arabidopsis as the most drought-responsive species (16% DEGs), followed by rice and barley (12% DEGs). Wheat had the lowest percentage (4%) of DEGs, which may be to the outcome of partial representation of transcripts on the Affymetrix array. Completion of the wheat genome sequence will facilitate the discovery of additional and novel drought-adaptive DEGs. Notably, the percentages of the identified DEGs were not associated with the different number of studies (r = −0.18, *P* = 0.82), and therefore reflect true differences between species.

### Gene ontology characterization in each species

The significant DEGs, in each species, were subjected to gene ontology (GO) enrichment analysis for functional characterization of their biological processes (Additional file 4: Figure S2). The highest number of significantly enriched biological-processes was found in Arabidopsis (663), followed by rice (180), wheat (86) and barley (27) (Figure 2B and C and Table 1). Strikingly, 81% of the biological-processes detected in Arabidopsis were species-specific while rice, wheat and barley had only 48%, 34% and 7% of species-specific enriched biological-processes, respectively (Figure 2B and C). The substantial differences in the number and uniqueness of the GO biological-processes in each species may reflect the considerable lag in research and gene annotations that characterizes crop-plants.

To test the ability of the meta-analysis to identify new biological processes, we compared Arabidopsis GO list, obtained by the meta-analysis, with a subset of three original GO lists, obtained from WT Arabidopsis studies included in the meta-analysis. Interestingly, only 34% similarity was observed (Additional file 5: Figure S3), and all common biological-processes, found among the three individual lists, were also detected by the meta-analysis approach. The ability of the meta-analysis approach to detect additional 66% biological-processes demonstrates its analytic power to reveal new pathways that have been overlooked by individual studies.

### Identification of drought-adaptive genes using cross-species meta-analysis

A comparative platform across-species was developed by combining the fold-change scores obtained for each gene in the meta-analysis. To accomplish this, an injective (one-to-one) orthology relationship was defined, using the Model Genome Interrogator (MGI) and predicted orthologs among the four species were identified. The rice database was used as a reference for all species due to the high number of orthologs detected compared with Arabidopsis (9,104 *vs*. 4,939 for rice and Arabidopsis orthologs, respectively; Additional file 6: Table S3). The transformation to rice orthologs reduced dramatically the number of detected genes. From a total of 15,953 detected genes across the four species in the meta-analysis (Table 1 and Additional file 3: Table S2), 8,471 orthologs remained (53%; Additional file 6: Table S3), of which 5,520 orthologs belong to rice. A prominent reduction in gene number was observed for Arabidopsis and wheat (73% and 74% loss, respectively) followed by barley (49%) and rice (25%). The reduced number of wheat orthologs could result from an incomplete database, which may explain the substantial difference between the number of orthologs common to rice and barley (264 genes) compared with the number of orthologs common to rice and wheat (83 genes). It may also account for the low number of orthologs (28 genes) present in all three monocots (Figure 2D and E and Additional file 7: Table S4). In Arabidopsis, the reduced number of orthologs could also be explained by the high evolutionary distance from rice (i.e. eudicot *vs*. monocots).

Another analytical challenge in combining datasets of various species is to overcome species-specific residual variation in fold-change and substantial differences in database size. Penalized Fisher method was used to combine *P*-value distributions from each species meta-analysis. Significant cross-species DEGs were detected using adjusted *P*-value cutoff of 0.05 without setting a cross-species fold-change threshold. The advantage of this analytical setup is its improved ability to detect genes with consistent expression differences across taxa, which may have been overlooked due to their mild expression change. This approach resulted in identification of 225 DEGs across-species, comprised of 162 up-regulated (Average FC = 1.42, SD_FC_ = 0.20) and 63 down-regulated (Average FC = 1.38, SD_FC_ = 0.17) shared orthologs (Table 2 and Additional file 8: Table S5).

**Table 2:** **Functional classification of the shared drought-adaptive DEGs across-species**

**General category**	**Main functional category**	**Rice genes and their Arabidopsis orthologs as predicted by MapMan and BLAST2GO**	
		**Up-regulated**	**Down-regulated**
**Regulatory functions**			
RNA regulation	Transcription regulation	loc_os02g02390 (AT1G12800, S1 RNA-binding domain-containing protein), loc_os06g35960 (AT3G24520, HSFC1), loc_os05g38820 (AT2G37060, nuclear factor yb2)	loc_os12g42610 (AT2G26580, YAB5), loc_os03g08790 (AT1G09750, chloroplast nucleoid DNA-binding protein-related)
	RNA binding, transcription	loc_os03g17060 (AT2G37510, RNA-binding), loc_os03g44484 (AT4G21710, NRPB2), loc_os08g30820 (AT4G29820, CFIM-25)	
Signaling	Calcium		loc_os02g03020, loc_os06g46950 (AT2G46600, calcium-binding protein), loc_os03g20370 (AT2G27030, CAM5)
	Light	loc_os03g10800 (AT2G14820, NPY2), loc_os07g08160 (AT3G22840, ELIP1)	
	G-proteins and miscellaneous	loc_os03g05280 (AT5G03530, RAB ALPHA), loc_os07g33850 (AT5G54840, SGP1),loc_os07g44410 (AT4G01870, tolB protein-related)	
Protein	Degradation	loc_os01g12660 (AT1G64110, DAA1), loc_os01g52110 (AT5G25560, zinc finger family protein), loc_os04g45470, loc_os02g43010 (AT1G62710, β-VPE), loc_os08g38700 (AT1G55760, BTB/POZ domain-containing protein), loc_os02g02320 (AT3G10410, scpl49), loc_os02g27030 (AT4G39090, RD19), loc_os05g44130 (AT1G78680, GGH2), loc_os06g21380 (AT3G57680, peptidase S41 family protein), loc_os11g26910 (AT5G42190, ASK2), loc_os02g13140 (AT4G29490, aminopeptidase), loc_os03g54130 (AT5G45890, SAG12), loc_os05g35110 (AT1G21410, SKP2A)	loc_os12g24390 (AT3G54780, zinc finger family protein), loc_os06g03580 (AT3G63530, BB), loc_os02g48870 (AT5G10770, chloroplast nucleoid DNA-binding protein)
	Postranslational modification	loc_os03g27280 (AT1G78290, SNRK2.8), loc_os01g40094 (AT1G17550, HAB2), loc_os01g64970 (AT1G10940 ,SNRK2.4), loc_os01g10890 (AT5G45820, CIPK20), loc_os01g35184 (AT4G24400, CIPK8), loc_os09g25090 (AT5G25110, CIPK25), loc_os12g02200 (AT5G07070, CIPK2), loc_os06g08280 (AT3G46920 ,protein kinase family protein)	loc_os01g70130 (AT5G50860, protein kinase family protein), loc_os05g51420 (AT5G62740, HIR1)
	Folding and targeting	loc_os06g02380 (Chaperonin-60BETA2), loc_os12g02390 (AT3G52850, VSR1)	
	Synthesis	loc_os05g31020 (AT1G12920, ERF1-2), loc_os05g51500 (AT1G76810, elF-2 family protein)	
Chromatin structure	Histone	loc_os01g05630 (AT5G22880, H2B)	
Development	LEA protein, unspecified	loc_os06g23350 (AT3G22490, LEA protein), loc_os05g46480 (LEA3), loc_os03g21060 (AT1G69490, NAP), loc_os12g41680 (AT1G56010, NAC1), loc_os02g53320 (AT3G03270, USP family protein), loc_os04g43200 (AT2G33380, RD20), loc_os01g66120 (AT1G01720, ATAF1), loc_os03g26870 (AT1G78070, Transducin/WD40 repeat-like superfamily protein)	loc_os12g32620 (AT1G10200, WLIM1), loc_os09g36600 (AT4G34950, nodulin family protein)
Hormone metabolism	Abscisic acid	loc_os02g52780 (AT3G19290, ABF4), loc_os03g57680 (AT5G20960, AAO1), loc_os05g49440 (AT1G05510)	
	Gibberelic acid		loc_os06g15620 (AT1G74670, GASA6), loc_os03g42130 (AT3G19000, oxidoreductase)
	Ethylene	loc_os01g32780 (AT1G68300, USP family protein), loc_os12g36640 (AT2G47710, UPS family protein), loc_os01g51430 (AT2G26070, RTE1)	
**Response to stimulus**			
Abiotic and biotic stress	Heat, drought	loc_os02g32520 (AT5G51070, ERD1), loc_os05g44340 (AT1G74310, HSP101), loc_os03g16030, loc_os01g04380 (AT5G59720, HSP18.2), loc_os03g11910 (AT2G32120, HSP70T-2), loc_os03g31300 (AT5G15450, APG6), loc_os05g38530 (AT3G12580, HSP70), loc_os11g47760 (AT5G02500,HSP70.1), loc_os11g26760 (dehydrin Rab16C)	loc_os02g04120 (AT2G18250, COAD), loc_os04g33060 (AT1G32220,dehydratase family protein)
	Signaling	loc_os02g10350 (AT4G02600, MLO1)	
	Unspecified and biotic stress	loc_os06g40120 (AT5G20150, SPX1), loc_os03g18850 (PR1), loc_os11g10480 (AT1G77120, ADH1)	loc_os08g35760 (AT5G20630, GLP3), loc_os04g38450 (AT4G39640 ,GGT1), loc_os01g28500 (AT2G14610, PR1)
Biodegradation of Xenobiotics		loc_os01g47690 (AT1G53580, GLX2-3), loc_os06g20200 (AT5G23530, CXE18)	
**Localization & organization**			
Transport	TIP/NIP		loc_os03g05290 (AT2G36830, TIP1;1), loc_os06g22960 (AT3G16240, TIP2;1), loc_os10g36924 (AT4G10380, NIP5;1), loc_os06g12310 (AT5G37820, NIP4;2)
	Sugars	loc_os02g17500 (AT1G67300, hexose transporter)	loc_os07g39350, loc_os03g10090 (AT3G18830, ATPLT5), loc_os07g01560 (AT1G11260, STP1)
	Amino acids	loc_os02g54730 (AT2G41190, amino acid transporter family protein)	loc_os07g04180 (AT5G49630, AAP6)
	Nitrate		loc_os04g40410 (AT5G50200, NRT3.1)
	Peptides and misc.	loc_os05g32630 (AT3G05290, PNC1), loc_os08g06010 (AT3G47420, G3PP1), loc_os03g43720 (AT3G13050, NIAP), loc_os02g39930 (AT5G58070, ATTIL), loc_os04g36560 (chloride channel)	loc_os10g22560 (AT2G02040, PTR2-B), loc_os04g57200 (metal ion transport)
Metal handling	Metal binding	loc_os04g17100 (AT5G66110, metal ion binding), loc_os04g32030 (AT5G50740, metal ion binding)	
Cell	Organization	loc_os07g37560 (AT1G50360, VIIIA)	loc_os07g38730 (AT5G19780, TUA5)
	Death	loc_os03g05310 (AT3G44880, ACD1)	
**Energy**			
Mitochondrial electron transport	Electron transfer flavoprotein	loc_os04g10400 (AT5G43430, ETFBETA), loc_os03g61920 (AT1G50940 ETFALPHA)	
	Cytochrome c reductase	loc_os02g33730 (AT1G15120, ubiquinol-cytochrome C reductase complex 7.8 kDa protein)	
Photosynthesis	Light reaction and Calvin cycle	loc_os01g12710 (AT4G13250, SDR family protein)	loc_os07g05360 (AT1G79040, PSBR), loc_os11g47970 (AT2G39730, RCA), loc_os05g22614 (AT3G46780, PTAC16)
**Metabolic processes**			
Carbohydrate metabolism	Starch synthesis and degradation	loc_os05g50380 (AT1G27680, APL2), loc_os07g22930 (AT1G32900 , Starch synthase), loc_os03g04770 (AT3G23920, BAM1), loc_os09g29404 (AT4G09020, ISA3)	loc_os10g40640 (AT4G16600, transferase)
	Sucrose synthesis and degradation	loc_os08g20660 (AT5G20280, SPS1F), loc_os04g33490 (AT5G22510, INV-E), loc_os02g01590 (AT1G12240 , VAC-INV), loc_os05g45590 (AT4G29130, ATHXK1), Loc_os09g33680 (AT1G02850, BGLU11)	
	Raffinose and galactinol synthesis	loc_os07g48830 (AT1G56600, AtGolS2), loc_os03g20120 (AT2G47180, AtGolS1) loc_os08g38710 (AT1G55740, AtSIP1)	
	Galactose metabolism	loc_os10g35070 (AT5G08380, AGAL1), loc_os07g48160 (AT3G56310, AGAL putative), loc_os01g33420 (AT3G26380, AGAL putative), loc_os05g51670 (AT4G10960, UGE5)	loc_os04g38530 (AT5G15140, Galactose mutarotase-like superfamily protein)
	Trehalose synthesis	loc_os10g40550 (AT4G22590, TPPG), loc_os02g44230 (AT5G51460, TPPA)	
	Miscellaneous	loc_os03g45390 (AT1G64760, glycosyl hydrolase family 17 protein), loc_os03g15020 (AT2G28470, BGAL8), loc_os07g23880 (AT3G23640, HGL1)	
Amino acid metabolism	Synthesis	loc_os05g38150 (AT2G39800, P5CS1), loc_os04g52450 (AT3G22200, GABA-T)	loc_os04g33390 (AT1G08250, ADT6), loc_os07g42960 (AT1G22410, DAHP synthase), loc_os03g63330 (AT5G13280, AK1)
	Degradation	loc_os05g03480 (AT3G45300, IVD), loc_os03g44150 (AT5G46180, Δ-OAT), loc_os06g01360 (AT5G54080, HGO), loc_os05g39770 (AT3G08860, PYD4)	loc_os04g53230 (AT1G11860, aminomethyltransferase), loc_os04g43650 (AT1G08630, THA1)
	Miscellaneous		loc_os04g20164 (AT4G12290, amine oxidase)
Polyamine metabolism	Spermidine synthase	loc_os06g33710 (AT5G53120, SPDS3)	
TCA\organic transformation	Organic acid transformaitons, carbonic anhydrases	loc_os02g07760 (AT1G79440, ALDH5F1), loc_os09g28910 (AT4G33580, BCA5), loc_os01g11054 (AT3G14940, ATPPC3)	loc_os04g33660 (AT3G52720, ACA1)
Fermentation	Aldehyde dehydrogenase	loc_os09g26880 (AT1G54100 ,ALDH7B4), loc_os08g32870 (AT1G74920, ALDH10A8)	loc_os02g43194 (AT4G36250, ALDH3F1)
	Pyruvate decarboxylase	loc_os01g06660 (AT4G33070, PDC1)	
Lipid metabolism	Synthesis	loc_os11g05990 (AT3G11670, DGD1), loc_os09g21230 (AT5G23050, AAE17), loc_os12g04990 (AT5G27600, LACS7), loc_os01g57420 (AT2G20900, DGK5), loc_os10g39810 (AT4G12110, SMO1-1)	
	Degradation	loc_os09g37100 (AT4G35790, PLDDELTA), loc_os07g47250 (AT5G18640, lipase class 3 family protein), loc_os07g47820 (T3G06810, IBR3), loc_os11g39220 (AT5G65110, ACX2), loc_os10g04620 (AT5G16120, hydrolase), loc_os03g07180 (embryonic protein DC-8)	loc_os03g40670 (AT5G08030, GDPD6)
	Desaturation, transfer	loc_os11g24070	loc_os03g18070 (AT3G11170, FAD7)
Secondary metabolism	Isoprenoids	loc_os02g07160 (AT1G06570, PDS1), loc_os01g02020 (AT5G47720, AACT1)	loc_os02g04710 (AT2G07050, CAS1)
	Phenylpropanoids and misc.	loc_os07g42250 (AT3G51420, SSL4)	loc_os04g15920 (AT4G39330, CAD9), loc_os11g32650 (AT5G13930, CHS)
Tetrapyrrole synthesis	Glutamyl-tRNA reductase		loc_os10g35840 (AT1G58290, HEMA1)
Nucleotide metabolism	Synthesis, adenine salvage	loc_os05g49770 (AT3G12670, emb2742)	loc_os02g40010 (AT1G80050, APT2)
	Degradation	loc_os02g50350 (AT3G17810, PYD1), loc_os08g13890 (AT1G67660, exonuclease)	loc_os04g58390 (AT4G04955, ALN)
Cell wall	Modification		loc_os06g48200 (AT5G57550, XTR3), loc_os01g60770 (AT1G69530, EXPA1), loc_os10g40720 (AT1G65680, ATEXPB2), loc_os05g39990 (AT2G40610, ATEXPA8)
	Degradation	loc_os09g31270 (AT3G57790, Pectin lyase-like superfamily protein), loc_os03g53860 (AT5G20950, glycosyl hydrolase family protein),	
Redox	Ascorbate, glutathione	loc_os12g29760 (AT4G33670, L-GalDH)	loc_os02g44500 (AT4G11600, GPX6)
	Heme	loc_os02g33020 (AT3G10130, SOUL heme-binding family protein	
	Miscellaneous	loc_os03g16210 (AT5G06060, tropinone reductase),	loc_os03g04660 (AT4G39490, CYP96A10), loc_os07g48020 (AT5G05340, peroxidase), loc_os07g48050 (AT5G05340, peroxidase)
Unspecified processes		loc_os03g17470 (AT3G55040, GSTL2), loc_os01g08440 (AT4G15550, IAGLU), loc_os01g05840 (AT2G37540, SDR family protein), loc_os02g51930 (AT1G22400, UGT85A1), loc_os10g40570 (AT1G63370, FMO family protein), loc_os12g21789 (AT3G49880, glycosyl hydrolase family protein 43), loc_os11g03730 (AT3G10740, ASD1), loc_os06g22080 (AT3G51520, diacylglycerol acyltransferase family), loc_os06g49990 (AT3G51130)	
**Unclassified**			
		loc_os10g32680 (AT1G07040), loc_os11g37560 (AT3G55760), loc_os01g46600 (PM41), loc_os03g51350, loc_os01g40280 (AT5G35460), loc_os09g20930, loc_os03g45280 (dehydrin), loc_os04g34610 (AT1G43245), loc_os03g48380 (AT1G27150), loc_os08g33640 (AT1G23110), loc_os01g58114 (AT4G27020), loc_os05g33820 (AT1G10740), loc_os02g48630 (AT5G48020), loc_os05g48230 (AT4G13400), loc_os09g04100 (AT4G31830), loc_os01g26920 (AT2G39080)	loc_os02g38240 (AT4G24750), loc_os07g12730 (AT5G01750)

To compare the CSA:Drought results to the original experiments included in the meta-analysis we examined two case studies using Arabidopsis and wheat experiments (Additional file 9: Figure S4). Among the 225 shared DEGs, only five genes (two genes involved in proteolysis, two genes encoding transporters and one gene associated with purine catabolism) were also reported among all three Arabidopsis studies [5,26,27]. The majority (62%) of the shared drought-adaptive DEGs were not reported in any of these experiments (Additional file 9: Figure S4A and Additional file 10: Table S6). This pattern was even more prominent among wheat studies [28-30], where none of the shared DEGs was detected by all three individual studies. Moreover, 82% of the shared DEGs were not reported in any of the three wheat studies (Additional file 9: Figure S4B and Additional file 10: Table S6). Remarkably, a higher number of overlapping genes was detected among the three individual Arabidopsis experiments (e.g. 46 genes present in all three studies). These common DEGs may imply Arabidopsis specific adaptations to drought stress rather than general plant drought adaptations.

### Metabolic pathway analysis of shared drought-adaptive DEGs

**Figure 3:**
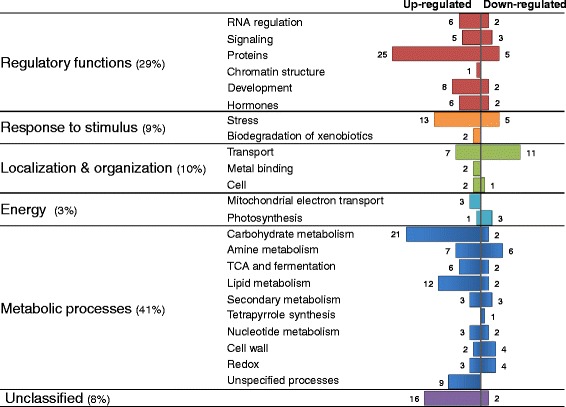
Functional classification of shared drought-adaptive DEGs based on MapMan and BLAST2GO annotations.

The 225-shared drought-adaptive DEGs were further analyzed for their associated GO biological-process terms and functional categories. GOs describe gene products in a species-independent manner [31], making it a useful functional classification for cross-species comparisons. REVIGO clustering highlighted response to abiotic stimulus and carbohydrate metabolism among up-regulated biological processes, whereas, metabolism of amines and aromatic compounds, and transport were included among down-regulated biological processes (FDR ≤ 0.05) (Additional file 11: Table S7). To complement this approach, the 225-shared drought-adaptive DEGs were analyzed for their corresponding functional categories based on the species-specific MapMan annotations. Additional effort to minimize the number of DEGs with unknown function or classification was undertaken using the BLAST2GO program (Figure 3 and Table 2).

The largest functional group (41%) of DEGs was associated with metabolic processes (e.g. metabolism of lipids, nucleotides, secondary metabolites and cell wall), suggesting a considerable rearrangement in plant metabolism as part of progressive drought adaptation. Thirty-five of these genes were involved in carbohydrate and amino acid metabolism (e.g. up-regulation in synthesis of stress-related sugars such as raffinose, galactinol and trehalose and synthesis of proline and GABA). Several of these genes were shown to be involved in synthesis of osmoprotectants, which ameliorate the detrimental effects of drought (reviewed by [32]). Up to 29% of the shared DEGs were involved in putative regulatory functions (e.g. transcription regulation, signaling, protein degradation, post-translational modifications and hormones). The expression of genes involved in abscisic acid transduction and synthesis was found to be up-regulated, whereas genes associated with gibberellin biosynthesis and regulation exhibited down-regulation. Additional functional group of genes associated with response to stimulus (9%) was largely up-regulated (e.g. heat stress and xenobiotics degradation). Up-regulation of heat stress responsive genes was in accordance with up-regulation of heat-shock transcription factors. It is noteworthy, that 8% of the shared DEGs remained unclassified. These unassigned genes are intriguing since they hold the potential to contribute to drought adaptation and hence are novel drought-adaptive genes (Table 2).

### Promoter analysis of shared DEGs

To test whether putative regulatory regions, spanning DEG promoters, are enriched with *cis*-acting elements, across-species, DEG promoter motif enrichment analysis was conducted. Motif enrichment was limited to Arabidopsis and rice due to insufficient database support for wheat and barley. Significant motif enrichment was found only for the putative promoters of up-regulated DEGs. In Arabidopsis, three putative enriched motifs (Ga**CACGtg**, GA**CACGTg**TC and Ga**cACGTG**TC), found in 22 out of the 100 DEG promoters, are highly similar to the CACGTG core G-box motif (Additional file 12: Figure S5A). G-box was suggested to regulate gene expression in response to phytohormones and abiotic stimuli [33]. G-box motif can also be part of the ABA-Responsive Element (*ABRE*; ACGTGT), to which the two latter putative motifs are highly similar. In rice, three putative enriched motifs were identified (C**GCACGc,** T**GCGTG** and **gCGTG**CG; Additional file 12: Figure S5B) in 50 out of the 150 DEG promoters. The first motif (C**GCACGc**) is highly similar to a rice motif (GCACGC) that was enriched among dehydration inducible promoters [34]. The other two motifs contain the core sequence of Xenobiotic Response Element (*XRE*; GCGTG), which was found in promoters of animal genes, encoding xenobiotic metabolic enzymes [35], as well as in promoters of plant genes [36].

### Conservation analysis of drought-adaptive DEGs

**Figure 4:**
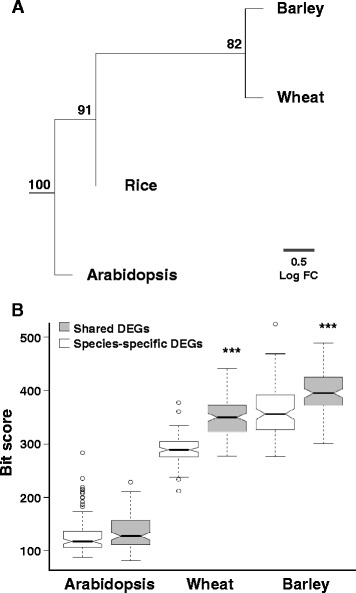
Conservation analysis. **(A)** Hierarchal clustering of pair-wise distance matrix based on expression profile of orthologs in each species. Bootstrap scores supporting the consensus tree (percentage) are indicated at each node. **(B)** Sequence conservation of shared DEGs *versus* species-specific DEGs. For each species, the bit score, obtained from the permutated blastn analysis, was compared between shared DEGs and species-specific DEGs. Bold horizontal bars indicate the average, boxes indicate the upper and lower 0.25 quartile, dashed bars indicate the max/min scores (excluding extremes), circles indicate the extremes, and notch overlaps indicate non-significant differences (*P* ≤ 0.05).

Functional and sequence conservation of the drought-adaptive DEGs across-species were further investigated by comparing the expression profiles and sequences of the identified DEGs. Due to substantial differences among species, only genes for which orthology could be determined in all four species were included in the analysis. A hierarchal clustering of pair-wise distance matrix, based on the expression fold-change in ortholog genes across species, recapitulated the known plant phylogeny (Figure 4A). Sequence conservation in shared *versus* species-specific DEGs was evaluated by comparing the corresponding sequences between the rice ortholog and each species (excluding a self-comparison for rice). For both shared and species-specific DEGs, higher sequence conservation was found among rice-barley and rice-wheat than for rice-Arabidopsis comparison (Figure 4B). Both functional and sequence conservation patterns found among species further support the CSA:Drought detection of cross-species DEGs. Significantly higher sequence conservation level of shared DEGs compared with species-specific DEGs, was found for barley (*t*_Welch_ = 5.91, *P* ≤ 0.0001) and wheat (*t*_Welch_ = 14.13, *P* ≤ 0.0001) (Figure 4B). The non-significant difference found in Arabidopsis, is presumably the consequence of the ample genetic distance between monocots and eudicots, indicated by a general lower sequence similarity and resolution.

### A case study of drought-adaptive genes in *Brachypodium distachyon*

**Figure 5:**
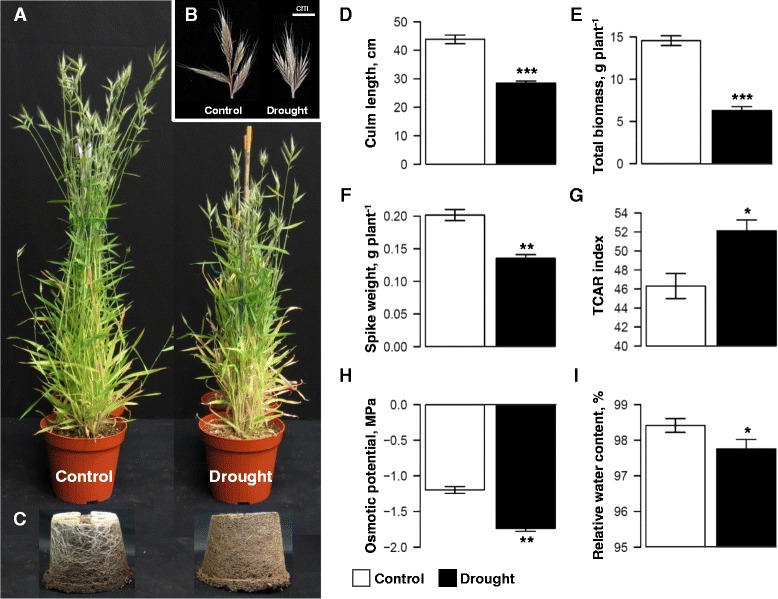
*Brachypodium distachyon* as a case study to validate the shared DEGs detected by CSA:Drought. **(A)** Plants grown under control and drought conditions. **(B)** Spike morphology, **(C)** Roots biomass, **(D)** Culm length, **(E)** Total biomass, **(F)** Spike weight, **(G)** Transformed chlorophyll absorbance in reflectance (TCAR) index, **(H)** Osmotic potential, and **(I)** Relative water content (RWC) under control and drought conditions. Values are mean ± SD (*n* = 5). *, ** and *** indicate significant differences between treatments at *P* ≤ 0.05, 0.01 and 0.001, respectively.

To validate the identified shared DEGs and evaluate their universality, we used the model grass *B. distachyon* [37] as a case study. Morpho-physiological characterization of plant adaptation to drought stress resulted in dramatic effects on plant growth (Figure 5A), spike morphology (Figure 5B) and root development (Figure 5C). Moreover, a significant reduction in culm length (*P* = 0.0001; Figure 5D), total biomass (*P* = 0.0001; Figure 5E) and yield production (*P* = 0.002; Figure 5F) was observed. Under drought stress, plants exhibited significant lower chlorophyll content (*P* = 0.02) based on transformed chlorophyll absorbance in reflectance index (TCARI; Figure 5G), higher osmotic potential (net solute accumulation in the cell: −1.19 ± 0.05 compared with −1.74 ± 0.04 for the control and drought treatment, respectively; Figure 5H) and a minor reduction in RWC (Figure 5I).

**Figure 6:**
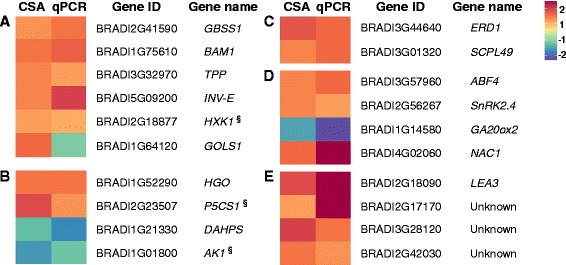
Heat-map of selected drought-adaptive genes detected by CSA:Drought and validated by qPCR analysis in *Brachypodium distachyon*. Red and blue represent high and low relative expression when compared to the mean value of expression across all samples, respectively. Scale is log_2_ of mean expression value. qPCR values, representing mean ± SD (*n* = 6), were calculated and normalized using *Glyceraldehyde 3-phosphate dehydrogenase* and *S-adenosylmethionine decarboxylase* as internal controls and presented as fold-change (*P* ≤ 0.05). **(A)** Carbohydrate metabolism: *GBSS1*, *Granule-bound starch synthase 1*; *BAM1*, *β-Amylase 1*; *TPP*, *Trehalose-6-phosphate phosphatase*; *INV-E*, *Alkaline/neutral invertase E*; *HXK1*, *Hexokinase 1*; *GOLS1*, *Galactinol synthase 1*. **(B)** Amino acid metabolism: *HGO*, *Homogentisate 1,2-dioxygenase*; *P5CS1*, *Delta1-pyrroline-5-carboxylate synthetase*; *DAHPS*, *3-Deoxy-D-arabino-heptulosonate 7-phosphate synthase*; *AK1*, *Aspartate kinase 1*. **(C)** Protein degradation: *ERD1*, *Early responsive to dehydration 1*; *SCPL49*, *Serine carboxypeptidase-like 49*. **(D)** Hormone metabolism and transcription factors: *ABF4*, *ABRE binding factor 4*; *SnRK2.4*, *SNF1-related kinase 2.4*; *GA20ox2*, *Gibberellin 20 oxidase 2; NAC1*, *NAC domain containing protein 1*. **(E)** Unknown and unclassified: *LEA3*, *Late embryogenesis abundant protein, group 3*. ^§^ indicates significant differences of qPCR analysis at *P* ≤ 0.1. Fold change values and statistical analysis for each gene can be found in Additional file 13: Figure S6.

A subset of 27 drought-adaptive DEGs, identified in the CSA:Drought, with various expression patterns, was selected for qPCR validation in *B. distachyon*. In general, this assay showed similar expression pattern as the CSA:Drought (except for *BdGOLS1*), with 20 significant genes (Figure 6, Additional file 13: Figure S6 and Additional file 14: Table S8). These genes included ***carbohydrate metabolic enzymes*** as *Granule-bound starch synthase 1* (*GBSS1*, regulator of amylose synthesis), *β-Amylase 1* (*BAM1*, involves in starch degradation), *Trehalose-6-phosphate phosphatase G* (*TPPG*, involves in trehalose synthesis), *Alkaline/neutral invertase E* (*INV-E*, hydrolyses sucrose into hexoses) and *Hexokinase 1* (*HXK1*, involves in hexoses catabolism and sugar signaling). Genes that encoded ***amino acid metabolic enzymes*** as *Homogentisate 1,2-dioxygenase* (*HGO*, involves in tyrosine degradation), *3-Deoxy-D-arabino-heptulosonate 7-phosphate synthase* (*DAHPS*, the first committed enzyme of the shikimate pathway), *Delta1-pyrroline-5-carboxylate synthetase* (*P5CS1,* the rate-limiting enzyme in proline biosynthesis) and *Aspartate kinase 1* (*AK1*, catalyzes the first reaction of lysine synthesis). Genes related to ***protein degradation*** as *Early responsive to dehydration 1* (*ERD1*, encodes a Clp protease regulatory subunit) and *Serine carboxypeptidase-like 49* (*SCPL49*, involves in proteolysis). ***Hormone metabolic enzymes*** and ***transcription factors***, including *ABRE binding factor 4* (*ABF4*, a bZIP transcription factor that mediates ABA-dependent stress responses), *SNF1-related kinase 2.4* (*SnRK2.4*, involves in osmotic stress responses and ABA signaling), *Gibberellin 20 oxidase 2* (*GA20ox2*, a key enzyme in gibberellin synthesis) and *NAC domain containing protein 1* (*NAC1*, involves in transcriptional regulation). Additionally, a random set of ***unknown function*** (*putative late embryogenesis abundant protein, group 3*, LEA3) and ***unclassified*** (BRADI2G17170, BRADI3G28120 and BRADI2G42030) genes were also analyzed.

The similar expression pattern, obtained in a fifth species that was not included in the CSA:Drought, reinforces the consistency of the shared DEGs as key genes involved in adaptation to progressive drought stress across-species (Figure 6).

## Discussion

Traditionally, comparisons between two contrasting water regimes were used to identify drought-related DEGs. This strategy yielded hundreds to thousands of DEGs, depending on the selected significance threshold, however, focus was predominantly given to genes with high fold-change (usually ≥ 2), overlooking functionally and biologically important genes with relative mild expression differences. Moreover, in most cases very limited overlaps were found among different studies. Our *working hypothesis* is that plant adaptation to drought stress involves combination of evolutionary conserved pathways, as well as, species-specific genes. Here we developed a novel cross-species meta-analysis platform to reveal a core set of shared genes and pathways by integrating transcriptional data from Arabidopsis, rice, wheat and barley into one meaningful analytical framework.

Most (75%) drought transcriptome studies have been conducted on Arabidopsis under artificial and extreme conditions (e.g. detached leaves and shocks) for short periods (e.g. minutes to hours) at the vegetative phase (e.g. young seedlings), with survival or recovery as selective traits. In addition, while functional analysis of candidate genes significantly improved drought resistance in transgenic lines under laboratory conditions, limited success was reported for transgenic crop-plants under field conditions [38], where crop-plants are often exposed to longer episodes of slowly developing drought stress [39]. Therefore, we focused our CSA:Drought strategy on progressive drought stress studies at the reproductive stage. This approach enabled detection of 225-shared drought-adaptive DEGs with enhanced functional and evolutionary conservation across-species (Figures 3, 4 and Table 2). Moreover, we were able to detect with the CSA:Drought approach 128 and 178 shared ortholog DEGs in Arabidopsis and wheat, respectively, that were missed by the original studies (Additional file 9: Figure S4). It is worth noted that while in Arabidopsis only treatment differed between studies (i.e. all studies conducted using Col-0 ecotype), in wheat both genotypes (e.g. genotypes Creso, Chinese Spring, Y12-3 and A24-39) and treatments differed, which may account for the limited overlaps compared with the shared DEGs. Additionally, in most cases, transcriptome analyses use arbitrary fold-change thresholds combined with significance levels to reduce the number of detected DEGs from few hundreds/thousands to a tractable subset. Such an approach highlights mostly species- and/or treatment-specific DEGs. In contrast, meta-analysis strategy facilitates detection of consistent and biologically important DEGs, which were overlooked in the original studies due to relatively low fold-change.

Relatively high level of sequence conservation was found among the shared DEGs compared with the species-specific DEGs (Figure 4B). This result should be considered in the light of the evolutionary distance between the four species and recent genetic bottlenecks involved in domestication and consciously evolution under domestication of rice, wheat and barley. It is worth notice that we cannot determine by our analysis if these genes were converged among species sometime during their separated evolutionary history. Although this seems unlikely, the sample size used in this study and the experimental design used in the original studies prevent us from completely rule out this option. Whether the sequence and functional similarity found among these genes is a consequence of conservation or convergence (or both), this shows that the shared DEGs play fundamental roles in drought adaptation.

Classification of the shared DEGs into functional categories suggests the involvement of various mildly expressed regulatory and metabolic pathways that jointly elicit an orchestrated drought adaptation (Figure 3 and Table 2). Among the metabolic processes carbohydrate and amine metabolisms are assigned as the largest sub-category (39%), which is involved in biosynthesis and accumulation of compatible solutes (Additional file 15: Figure S7). The functional conservation of these genes was demonstrated in an additional species. A randomly selected subset of 11 carbohydrate and amine metabolic *B. distachyon* orthologs showed similar expression pattern as CSA:Drought. In accordance, a higher osmotic potential was measured in drought stressed compared to control *B. distachyon* plants. Compatible solutes are small, nontoxic molecules that include sugars (maltose and trehalose), sugar alcohols (galactinol and mannitol), amino acids (proline) and amines (spermidine and glycine betaine) (reviewed by [40]). Compatible solutes are an important adaptive mechanism under drought stress as well as under additional abiotic stresses as salinity and extreme temperatures. Osmoprotectants facilitate maintenance of cell turgor and cellular water potential under stress, as well as acting in membrane and macromolecules stabilization and ROS scavenging (reviewed by [41]). Some of these osmoregulation-related shared genes have already been shown to improve drought tolerance. *TPPA* and *TPPG,* genes involved in trehalose synthesis, were included among up-regulated shared DEGs. Overexpression of yeast *TPS*-*TPP* in tobacco, Arabidopsis, rice and alfalfa significantly improved the transgenic plant drought tolerance [42-45]. Invertases mediate sucrose hydrolysis to glucose and fructose, which contributed to better osmoregulation [46]. Accordingly, *INV-E* was up-regulated under drought (Figure 6 and Additional file 13: Figure S6). Complex mechanisms operate in plants to coordinate the interactions between carbon assimilation and nitrogen metabolism [47]. Carbon and nitrogen balance is a key component in plant adaptation to drought stress [48]. Proline, synthesized via the glutamate pathway (*P5CS*), or from ornithine (*Δ-OAT*) [49], is believed to act as a store of carbon and nitrogen, as well as in ROS scavenging [50]. Both *P5CS1* and *Δ-OAT* expression levels were up-regulated under drought (Additional file 15: Figure S7). Accordingly, several studies have shown that overexpression of either *P5CS*, or *Δ-OAT*, in different plant species resulted in increased proline levels, which could contribute to enhanced stress tolerance [51-53]. Remarkably, among DEGs reported in studies included in the meta-analysis, only 16 osmoregulation-related shared genes were detected, with majority of these genes (10) present only in one study (Additional file 10: Table S6). It is worth noted that all Arabidopsis microarray experiments included in the meta-analysis overlooked the osmoregulation-related genes [5,26,27], and for other species only partial results were discussed [4,7,28-30,54]. Carbohydrate metabolism and lipid degradation may also be involved in supplying energy that is required for maintenance of drought adaptation and osmoprotectant synthesis through breakdown of energy reserves. Additional large group of genes were assigned to protein regulation and metabolism. Apart from its regulatory function, protein degradation during drought-induced leaf senescence results in increment of the free amino acid pool available for osmotic adjustment [48,55].

Phytohormone homeostasis is a key factor in plant drought adaptation that mediates a wide range of adaptive responses (reviewed by [1]). One of the fastest responses of plants to drought stress is synthesis of ABA, which induces gene expression, triggers stomata closure and eventually restricts cellular growth, leading to whole plant growth retardation. In accordance with ABA effects on reproductive tissue development, through transcriptional reprogramming [56] and ABA gene expression regulation during drought, which is mediated by transcription factors such as ABF4 (Figure 6), promoters of shared Arabidopsis orthologs were enriched with the cis-acting element ABRE (Additional file 12: Figure S5). ABRE involvement in ABA-regulated gene expression occurs after the accumulation of ABA and therefore many ABA-inducible transcription factors are involved mainly in late and adaptive drought processes [57]. Among the enriched ABRE genes included those involved in starch degradation and accumulation of compatible solutes [56], as detected by CSA:Drought and validated in *B. distachyon*, both transcriptionally and physiologically (Figures 5 and 6).

Interestingly, several genes that are known to regulate rapid drought-induced gene expression, were also detected by the CSA:Drought analysis. These genes included transcription factors as *SnRK2.4* and *SnRK2.8,* and a protease regulatory subunit as *ERD1*. Most drought-induced genes were detected under extreme drought conditions and short period assays, which might explain their annotations as early drought-responsive genes. However, the induction of these genes also during long, mild drought stress might imply on their involvement in maintenance of study-state gene expression level as part of drought adaptation. These discrepancies emphasize the importance of using physiologically oriented approach when designing stress assays.

## Conclusions

Our CSA:Drought strategy identified a set of 225 key drought-adaptive genes that were only partially, if at all, reported in the studies included in the meta-analysis. Functional categorization of the shared DEGs underlined various regulatory and metabolic pathways as conserved drought-adaptive mechanisms across species. Physiological and transcriptional characterization of drought stressed *B. distachyon*, further supported these results. Additionally, we have identified and validated a group of unclassified genes (8%) that could be further investigated of their functional prospective roles in drought adaptation mechanisms. The shared DEGs provide useful resource for subsequent research and can serve as a potential set of molecular biomarkers for drought experiments and as candidate genes for engineering drought-tolerant crop-plants.

## Methods

### Microarray meta-analysis

Raw microarray data files (.CEL) of progressive drought stress studies at the reproductive stage were obtained from Gene Expression Omnibus (http://www.ncbi.nlm.nih.gov/geo) and ArrayExpress (http://www.ebi.ac.uk/arrayexpress). Description of the obtained studies depicted in Additional file 1: Table S1. Both species-specific probe-set annotation file and the corresponding probe-gene maps were downloaded from the Affymetrix site (http://www.affymetrix.com). For each species, Affymetrix raw data files were converted and normalized in R (http://www.r-project.org) using the bioconductor ‘affy’ package [58]. Quality control analyses of the obtained microarrays included quantile normalization for each array, followed by across array robust multichip average (RMA) normalization and transformation to log_2_ scale.

Meta-analysis was conducted using the rank product statistics [59], which enabled to combine data of different origins and identify DEGs between treatment and control conditions. This non-parametric test was conducted over all replicates within species to decrease the residual effect of each study and increase statistical power to identify DEGs across experiments using the Bioconductor ‘RankProd’ package [60]. Briefly, genes are ranked based on their expression (up- or down-regulation) in response to drought in each experiment individually. The null hypothesis is that the order of genes in an experiment is random, hence the probability to detect a gene ranked among the top genes equals to its rank among the total number of genes in each experiment. For each gene a combined probability was calculated as the product of ranks across experiments and its significance was determined using 100 permutations to accurately estimate *P*-values [61]. DEGs were selected after correcting for multiple testing using the percentage of false-positive prediction, which also controls for the accumulated false positives with a cutoff of 0.05. For each species, DEGs heat-map was constructed using ggplot2 package [62]. To be able to compare between species, the number of detected DEGs was divided by the corresponding species array size.

### Gene ontology analysis

The DEGs were subjected to enrichment of gene ontologies (GOs) using the AgriGO toolkit (http://bioinfo.cau.edu.cn/agriGO). GO enrichment was based on the hypergeometric statistics followed by a 0.05 FDR correction for multiple comparisons with a minimum of five entities mapped to each category. The enriched GO biological processes were clustered and visualized using the web-server REVIGO (http://revigo.irb.hr). REVIGO clustering algorithm finds a single representative GO term, for clusters of semantically similar GO terms, thus resulting in reduced, non-redundant GO term sets (i.e. superclusters). The size of each supercluster reflects its *P*-value.

### Cross-species meta-analysis

We used the Model Genome Interrogator (MGI) tool in PLEXdb (http://www.plexdb.org) to retrieve predicted orthologs between each species and homologous loci in the rice model genome. The MGI matches one or more predicted orthologs to a selected microarray probe-set using GeneSeqer (parameters: −x 12 -y 16 -z 24 -w 0.2) followed by blastx to protein database and blastn to FL-cDNA sequence database (both with *E*-value < 1e-20), and back, producing a quality score for each pair. To define an injective (one-to-one) orthology between genes, only best alignment score for each probe-set-ortholog hit was considered. Shared DEGs were identified using the penalized Fisher method that combines the *P*-value distributions from all four species:

$$ {X}_g^2={\displaystyle \sum_{i=1}^k-2lo{g}_e\left({P}_{gi}\right)} $$

where *P*_*gi*_ is the probability that gene *g* was not differentially expressed between treatments (based on false-positive prediction)*.* This method could be affected by differences in dataset size between species, i.e. small *P*-values in one species may lead to subsequent small *P*-values in the cross-species combined distribution, as was detected for the non-normalized data (data not shown). Therefore, *P*-values were quantile normalized within each species prior to the penalized Fisher method. The combined *P*-values were further corrected using the FDR adjustment [63]. To enable the detection of significant items even when not present in all datasets, missing items from at most one dataset were included, dragging a *P*-value penalty equals to one instead of a missing value. Z-transform normalization was also examined, but was found to be sensitive to the use of penalty (not shown), due to summation compared with multiplication in the penalized Fisher method. For each DEG the average fold-change across-species was calculated using the geometric mean:

$$ {\overline{D}}_g= exp\left(\frac{1}{k}{\displaystyle \sum_{i=1}^klo{g}_e\left({D}_{gi}\right)}\right) $$

where *D* is the expression fold-change of gene *g* in species *i,* and *k* is the number of species from which the average fold-change was calculated.

### Metabolic-pathway analysis

DEGs were assigned to processes and pathways using MapMan software, which organizes genes in blocks, rather than as pathways. This designation allows genes to be tentatively assigned, even when their function is only roughly known [64]. Unassigned genes were further annotated with the program BLAS2GO (http://www.blast2go.com) using default parameters.

### Promoter analysis

Sequences of shared DEGs were extracted from Gramene BioMart (http://www.gramene.org) with 1 kb upstream to the transcription start site. Promoter analysis was conducted on the two model species Arabidopsis and rice, since wheat genome is not supported by BioMart, and approximately third of the barley gene sequences are not at adequate quality (i.e. < 800 bp or >200 N). Analysis of significantly overrepresented motifs within promoter sequences was conducted in BioProspector program [65] integrated in the Tmod software [66]. To model the base dependencies of each species, the second-order Markov background models were constructed based on a random sample of 100 and 150 promoters, which are equivalent to the size of up-regulated across-species genes in Arabidopsis and rice, respectively. Since several *cis*-acting elements, involved in plant responses to drought, e.g. ABA-responsive element (ABRE) and dehydration-responsive element (DRE), contain core hexamer sequences [67,68], a fixed motif width was set to 6 bp. For all other parameters the default settings were used and a null score was obtained based on the distribution of 100 Monte-Carlo simulations. The detected motifs, were further optimized and validated using the BioOptimizer program [69]. Logos were generated using WebLogo program (http://weblogo.berkeley.edu).

### Evolutionary analysis

To study the functional clustering of the four species, a pair-wise distance matrix was calculated using the expression profile of each species. The Euclidean distance between orthologs, as were determined by the Model Genome Interrogator and the following filtering procedure, was calculated using the expression fold-change in response to drought of all genes expressed across-species. A hierarchical clustering was conducted in R using a complete agglomeration of the pair-wise distance matrix and a phylogenetic tree was constructed after 100 bootstraps.

Shared DEGs were further analyzed for their DNA sequence conservation among the four species. For each shared DEG, the ortholog in rice was determined using the MGI tool and was used as a transitive anchor across species. The corresponding sequence in rice was obtained from the Rice Genome Annotation Project (http://rice.plantbiology.msu.edu) and mapped to the barley genome (Morex assembly [70]), wheat draft genome (LCG assembly [71]), and Arabidopsis genome (TAIR10; http://www.arabidopsis.org). The blastn program was used to compare all rice ortholog sequences to the other three species genomes with an *E*-value cut-off of *e*^−10^ and the bit-score was considered as a measurement for similarity between sequences. The use of bit-score enabled to reduce the bias introduced by the size of the searched database [72], which varies extensively between species. To avoid the residual variation introduced by gene duplication after speciation (paralogy), whole genome duplication (ohnology) or polyploidization (homeology) (in wheat), only the best hit (i.e. lowest *E*-value) was considered. The conservation of shared DEGs was further compared with DEGs uniquely detected in each species (i.e. species-specific DEGs). The ortholog sequences of unique DEGs in each species were obtained from the rice genome. A random sample of 50 genes was selected from each of the two DEGs lists of each species. The rice ortholog sequences were then compared to the corresponding species genome using blastn with same settings as previously described and the average bit score was recorded. This procedure was permutated 100 times with replacement and the average bit score over all samples was compared between the two DEG lists for each species using the Welch t-test.

### Physiological characterization of drought adaptation in *Brachypodium distachyon*

Seeds of *B. distachyon* accession 21–3 were obtained from the National Small Grains Collection (NSGC). Seeds were sown in trays containing soil mixture (Tuff Merom Golan, Israel) and stored in 4°C for 48 h followed by 5d in dark room (15°C). Seedling were transferred to greenhouse (22°C/16°C day/night, 10 h light/14 h dark) and planted in pre-weighted 1 L pots. Plants were fully irrigated three times a week and fertilized with 1 g L^−1^ N:P:K (20% nitrogen, 20% phosphorus, 20% potassium) + micronutrients, two months after germination. Plants were transferred to a long day regime (15 h light/9 h dark) 10 weeks after germination (six replicates in each treatment). At booting stage (BBCH scale 4.5 [73]) drought was applied gradually and maintained at 40% relative soil water content for 17d.

Measurements of osmotic potential and relative water content (RWC) were conducted on third leaf at mid-day. For osmotic potential analysis, leaves were placed in vials containing double-distilled water and kept in dark cold room for 4 h. Leaves were then dried and frozen in liquid nitrogen. Osmotic potential of the leaf sap was assessed using a vapor pressure osmometer (Vapro5600, Wescor Inc., USA). For RWC analysis, leaves were placed in pre-weighted vials. Vials were immediately weighted to obtain fresh weight (FW) followed by hydration for 6 h to full turgid. Samples were weighted to obtain leaf turgid weight (TW) and then oven dried at 75°C for 72 h to obtain dry weight (DW). RWC was calculated as:

$$ RWC=\left(FW\mathit{\hbox{-}}DW/TW\mathit{\hbox{-}}DW\right)*100 $$

Leaf spectral reflectance, at wavelengths from 400 to 1000 nm with an interval of ~0.2 nm, was measured at mid-day using a portable narrow-band width spectrometer (CI-700, CID Bio-Science Inc., USA). Leaf chlorophyll concentration was estimated using transformed chlorophyll absorption in reflectance index (TCARI) [74]:

$$ TCARI=3*\left[\left(W700-W670\right)-0.2*\left(W700-W550\right)*\left(W700/W670\right)\kern0.1em \right] $$

Culm length was measured from soil to spike base. Spikes and vegetative dry matter were harvested separately at the end of the experiment and oven dried (75°C for 72 h). Samples were weighed and total biomass was calculated.

### RNA extraction and qPCR assay

Flag and second leaf samples from six independent plants were collected in the morning of the 17^th^ day of drought stress and immediately frozen in liquid nitrogen. Total RNA was extracted using Plant/Fungi Total RNA Purification Kit (Norgen Biotek Corp., Canada) with on-column DNase treatment (Qiagen, Germany). RNA integrity was assessed with 2100 Bioanalyzer (Agilent Technologies Inc., Germany) and first strand cDNA was synthesized using qScript™ cDNA Synthesis Kit (Quanta Biosciences Inc., USA) following manufacturer’s instructions. qPCR was carried out using PerfeCTa® SYBR® Green FastMix® (Quanta Biosciences Inc., USA) on the PikoReal RT-PCR system (Thermo Fisher scientific Inc., USA). Gene-specific primers were designed using Primer-BLAST software [75] (Additional file 14: Table S8). The 2^-∆∆CT^ method [76] was used to normalize and calibrate transcript values relative to two housekeeping genes *Glyceraldehyde 3-phosphate dehydrogenase* (*GAPDH,* BRADI3G14120) and *S-adenosylmethionine decarboxylase* (*SamDC*, BRADI2G02580) [77], whose their expression did not change in response to drought.

### Availability of supporting data

The datasets supporting the results of this article are included within the article and its Additional files.

## Additional files

Additional file 1: Table S1. Summary of studies and arrays that were included in the CSA:Drought. Additional file 2: Figure S1. Hierarchal clustering of expression profiles in each species. Additional file 3: Table S2. Significant DEGs in each species, as calculated by rank product statistics. Additional file 4: Figure S2. Significant up- and down-regulated GOs in each species. Additional file 5: Figure S3. A comparison between the shared GOs detected by CSA:Drought and three independent Arabidopsis studies. Additional file 6: Table S3. Orthologs in each species, based on MGI tool. Additional file 7: Table S4. Common and unique orthologs found among the four species. Additional file 8: Table S5. Shared drought-adaptive DEGs across-species, based on penalized Fisher method. Additional file 9: Figure S4. A comparison between the shared DEGs and independent lists obtained from (A) Arabidopsis or (B) wheat studies included in the meta-analysis. Additional file 10: Table S6. Common genes between shared DEGs and independent lists obtained from Arabidopsis or wheat studies that were included in the meta-analysis. Additional file 11: Table S7. Significant up- and down-regulated shared biological processes across-species. Additional file 12: Figure S5. Enriched motifs in promoters of up-regulated shared drought-adaptive DEGs in (A) Arabidopsis and (B) rice. Additional file 13: Figure S6. Relative expression of shared drought-adaptive orthologs under controlled and drought stressed *Brachypodium distachyon* plants. Additional file 14: Table S8. List of primers used for the qPCR assay. Additional file 15: Figure S7. Alteration in expression of carbohydrate and amino acid metabolic genes, involved in osmoregulation under drought, that were detected by CSA:Drought.

## Abbreviations

ABA: Abscisic acid
ABRE: ABA-Responsive Element
BP: Biological process
DEG: Differentially expressed gene
FC: Fold-change
GO: Gene ontology
