# Supplementary material for: Identification of conserved drought-adaptive genes using a cross-species meta-analysis approach
Source: BMC Plant Biol. 2015 May 3;15:111. doi: 10.1186/s12870-015-0493-6 (PMC4417316; doi:10.1186/s12870-015-0493-6)
Supplement: Additional file 2: Figure S1. — Hierarchal clustering of expression profiles in each species. [file 12870_2015_493_MOESM2_ESM.pdf]

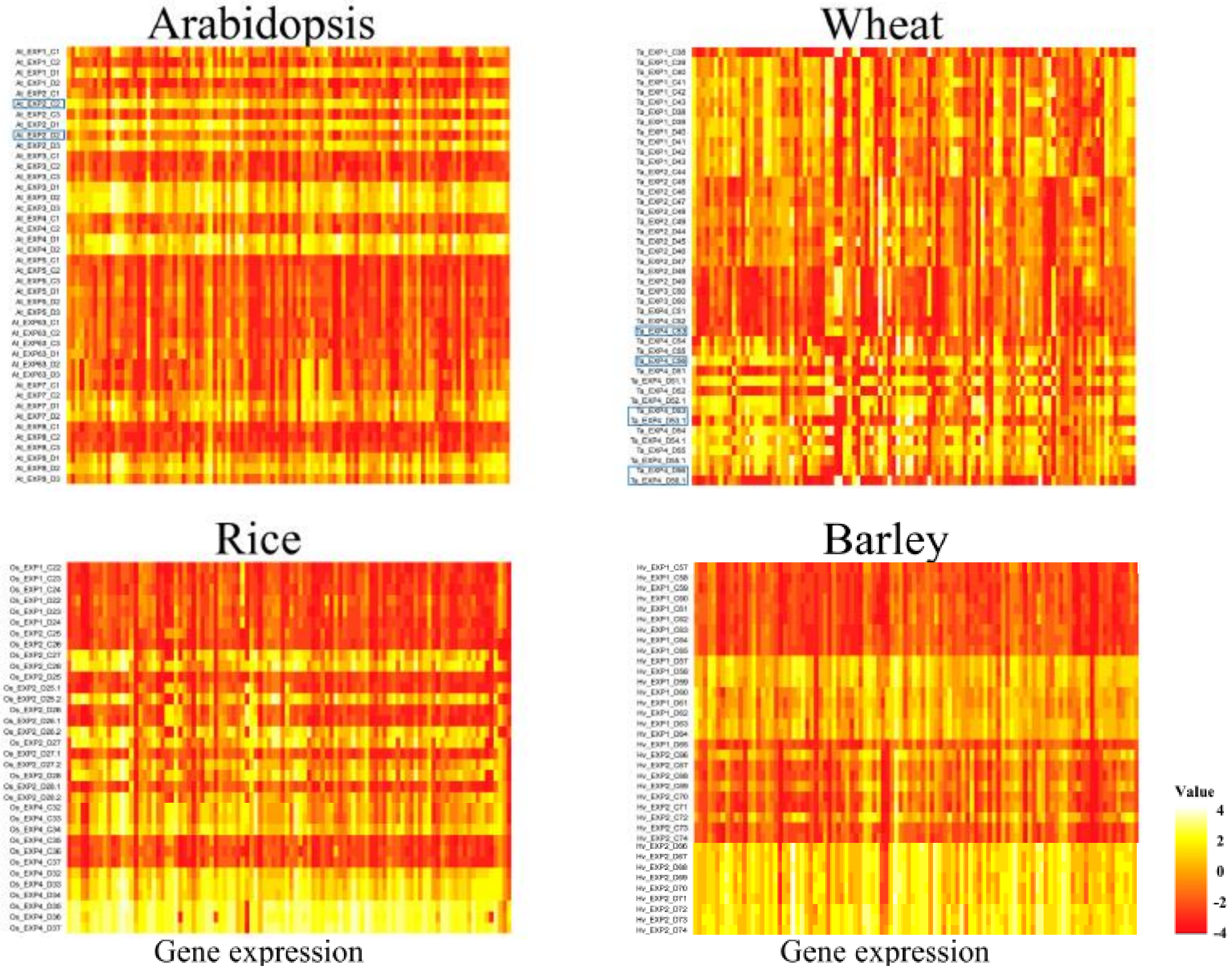

Additional file 2: Figure S1. Hierarchal clustering of expression profiles from all arrays and studies that were included in the meta-analysis in each species. Arrays marked with blue rectangles were omitted from meta-analysis due to inconsistent expression patterns.
