# Supplementary material for: Identification of conserved drought-adaptive genes using a cross-species meta-analysis approach
Source: BMC Plant Biol. 2015 May 3;15:111. doi: 10.1186/s12870-015-0493-6 (PMC4417316; doi:10.1186/s12870-015-0493-6)
Supplement: Additional file 4: Figure S2. — Significant up- and down-regulated GOs in each species. [file 12870_2015_493_MOESM4_ESM.pdf]

Arabidopsis up-regulated biological processes

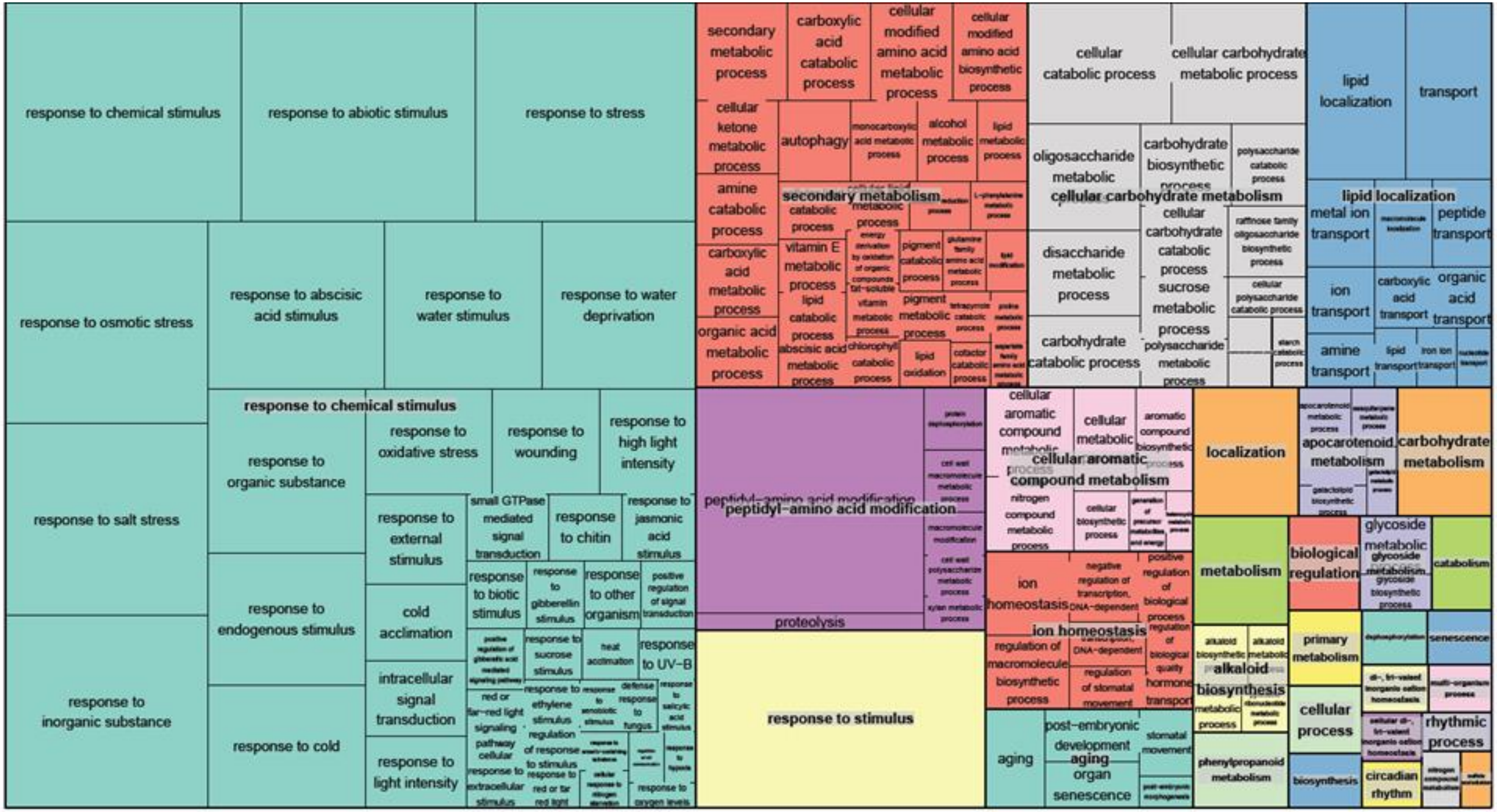

Additional file 4: Figure S2A. Significant up-regulated gene ontology biological processes (GO-BPs) in Arabidopsis (FDR ≤ 0.05), summarized and visualized by REVIGO.

Rice up-regulated biological processes

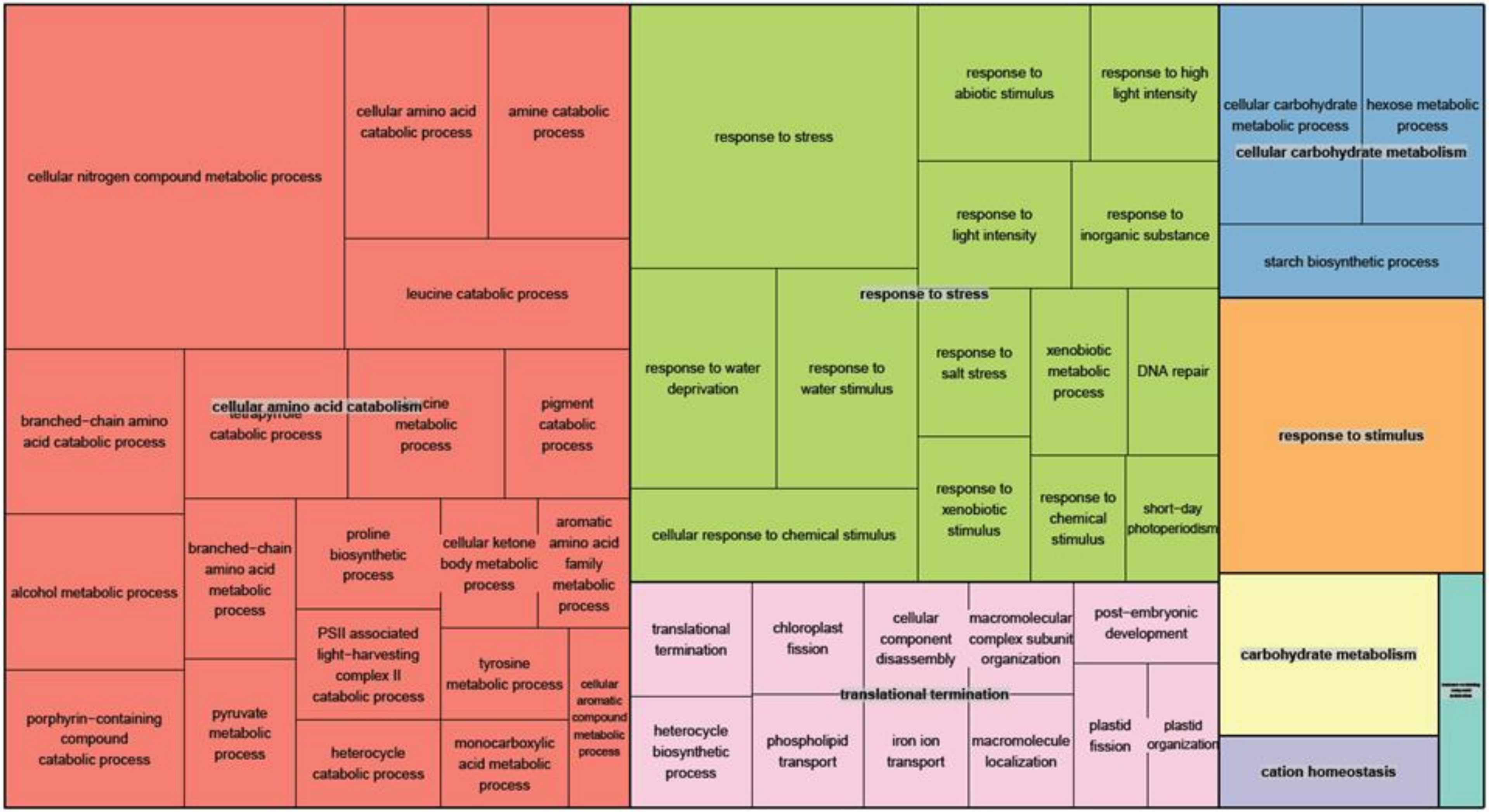

Additional file 4: Figure S2B. Significant up-regulated gene ontology biological processes (GO-BPs) in rice (FDR ≤ 0.05), summarized and visualized by REVIGO.

Wheat up-regulated biological processes

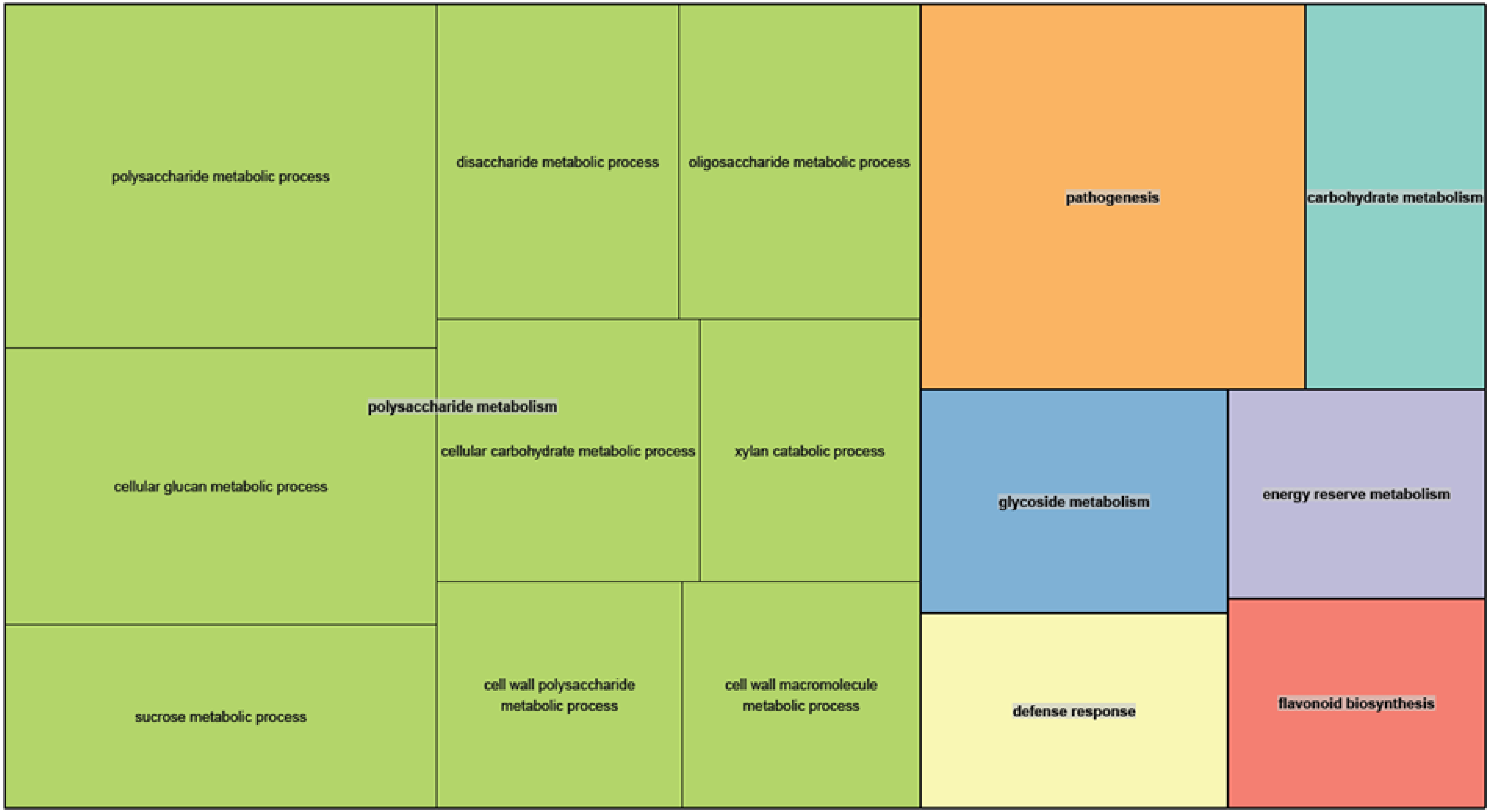

Additional file 4: Figure S2C. Significant up-regulated gene ontology biological processes (GO-BPs) in wheat (FDR ≤ 0.05), summarized and visualized by REVIGO.

Barley up-regulated biological processes

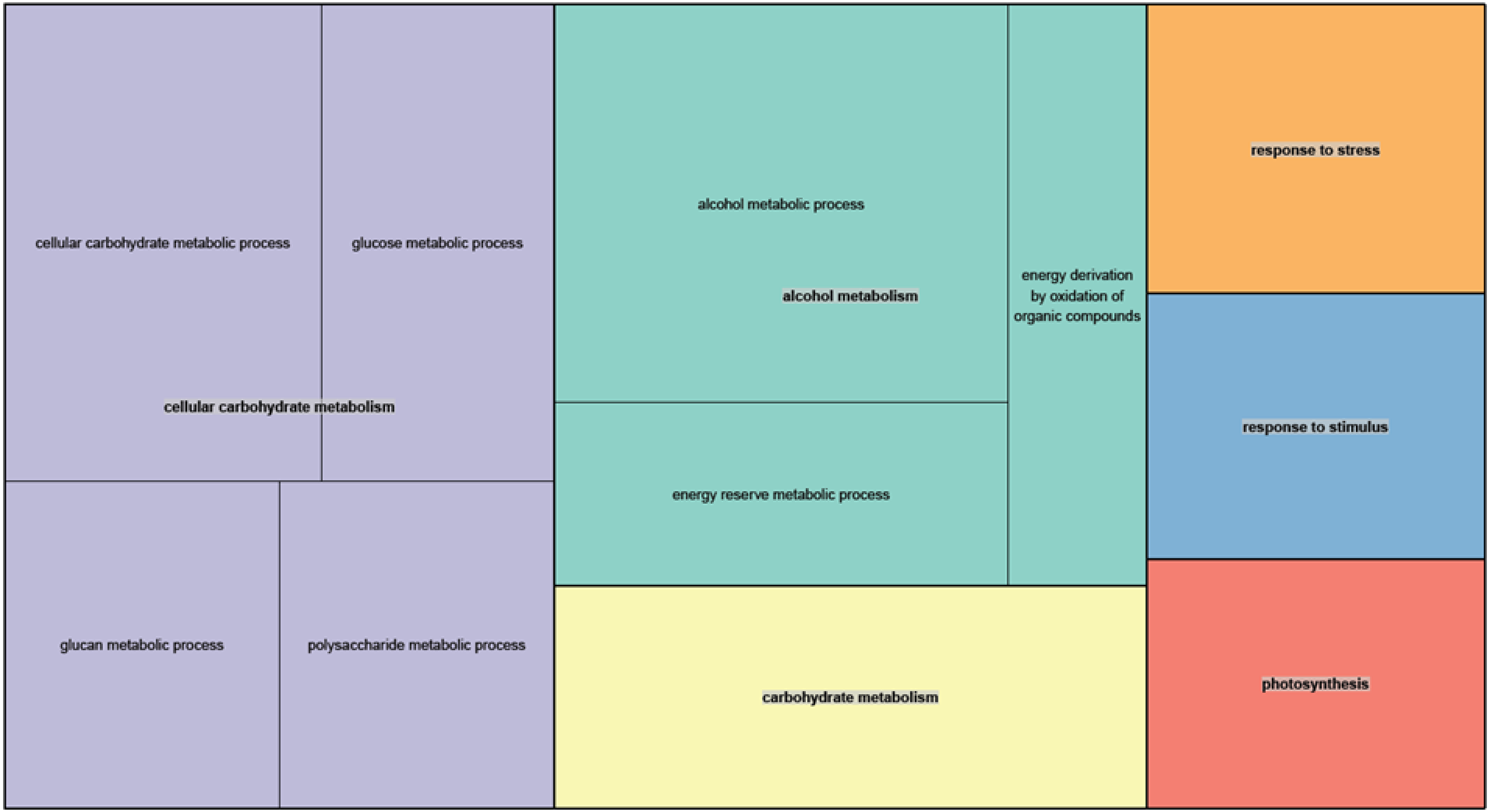

Additional file 4: Figure S2D. Significant up-regulated gene ontology biological processes (GO-BPs) in barley (FDR ≤ 0.05), summarized and visualized by REVIGO.

Arabidopsis down-regulated biological processes

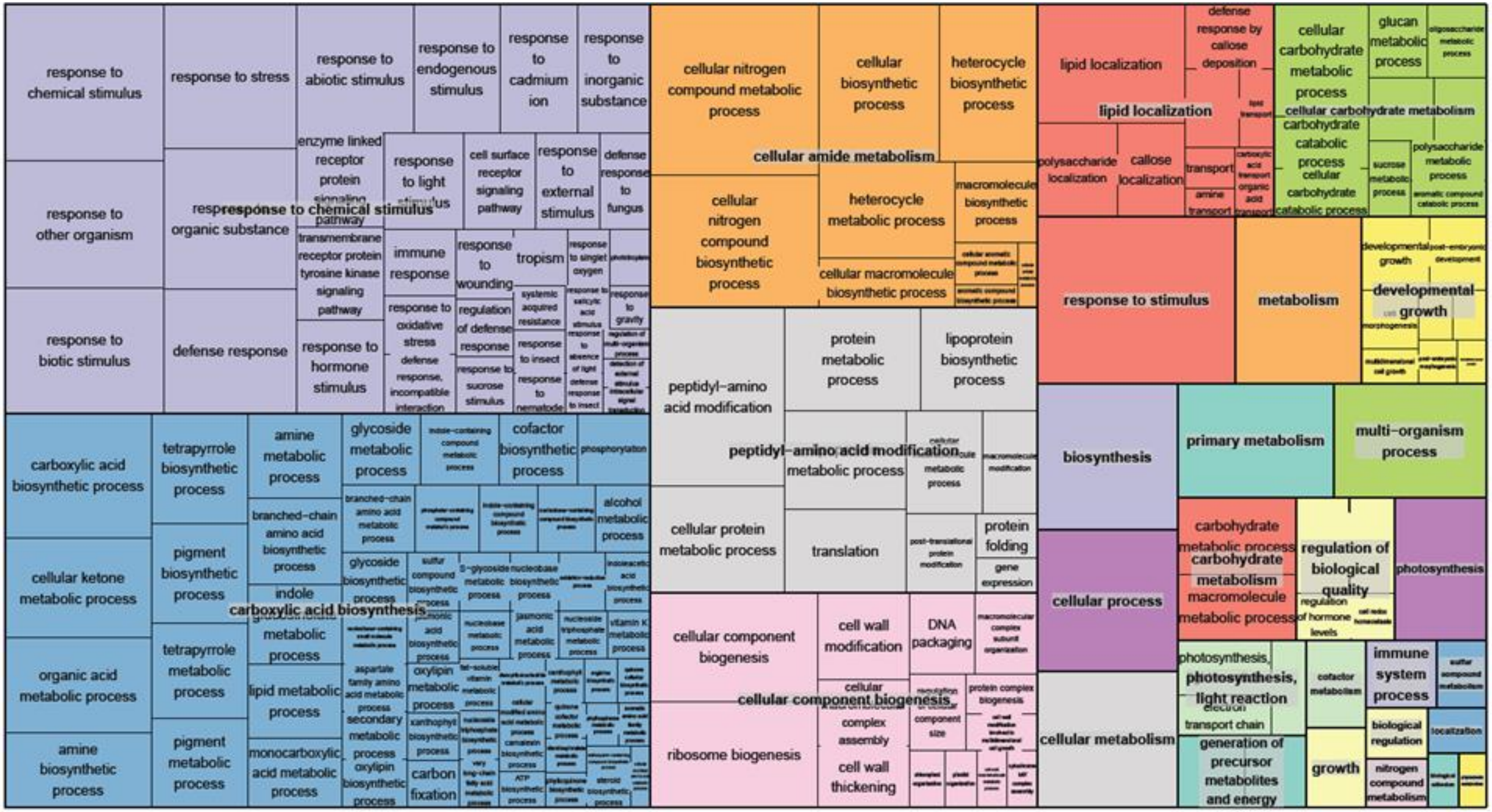

Additional file 4: Figure S2E. Significant down-regulated gene ontology biological processes (GO-BPs) in Arabidopsis (FDR ≤ 0.05), summarized and visualized by REVIGO.

Rice down-regulated biological processes

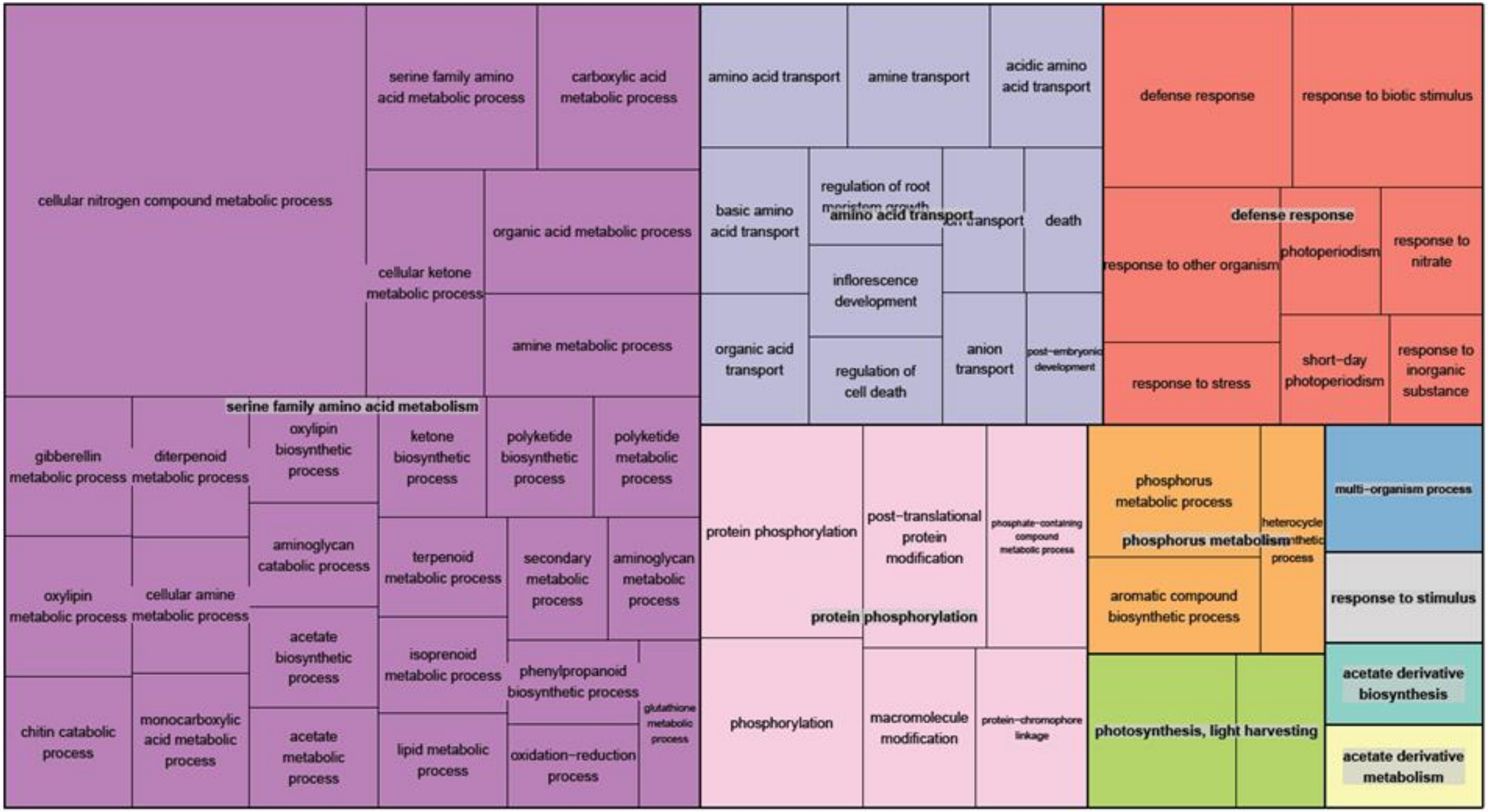

Additional file 4: Figure S2F. Significant down-regulated gene ontology biological processes (GO-BPs) in rice (FDR ≤ 0.05), summarized and visualized by REVIGO.

Wheat down-regulated biological processes

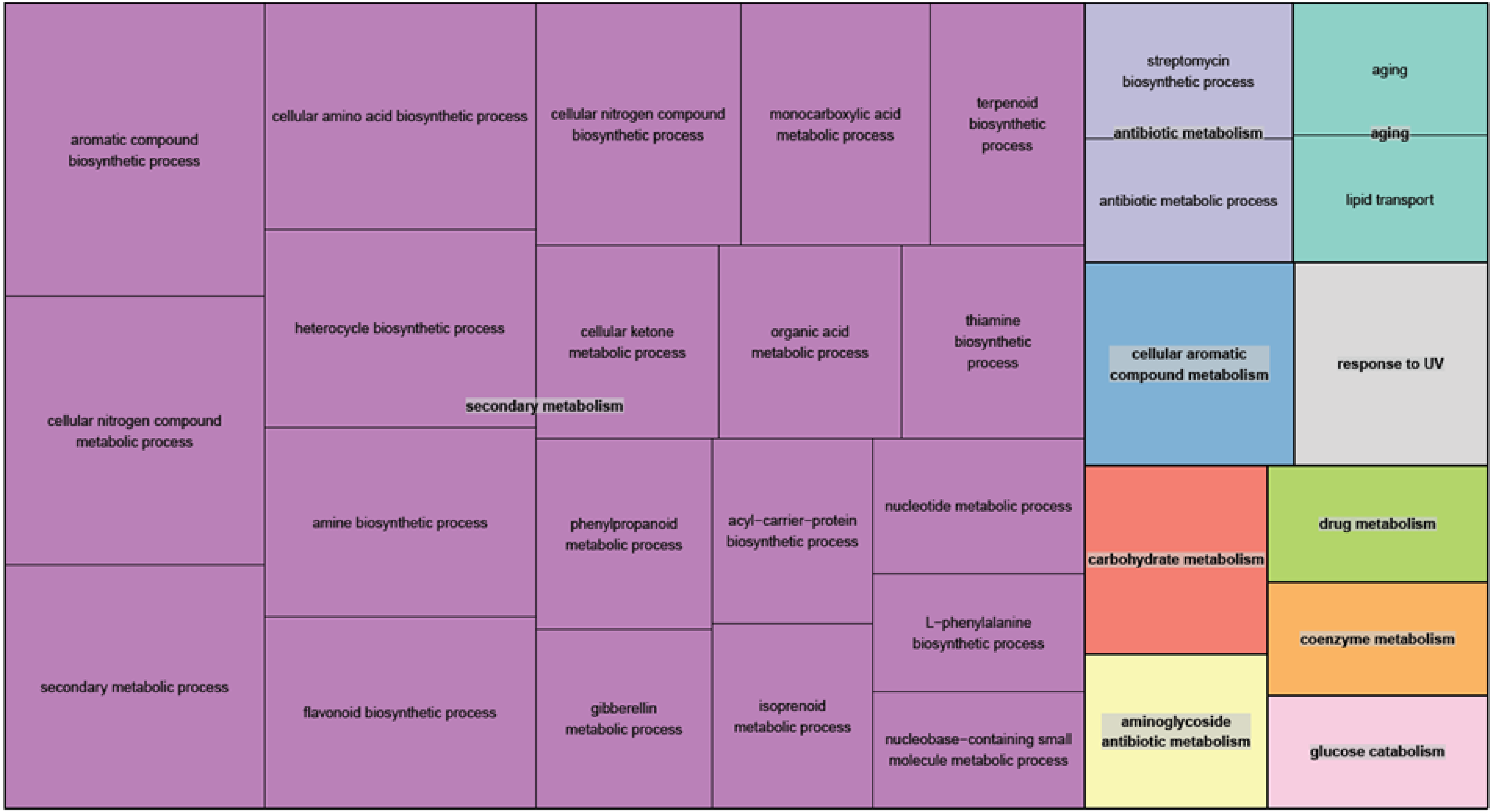

Additional file 4: Figure S2G. Significant down-regulated gene ontology biological processes (GO-BPs) in wheat (FDR ≤ 0.05), summarized and visualized by REVIGO.
