# Supplementary material for: Identification of conserved drought-adaptive genes using a cross-species meta-analysis approach
Source: BMC Plant Biol. 2015 May 3;15:111. doi: 10.1186/s12870-015-0493-6 (PMC4417316; doi:10.1186/s12870-015-0493-6)
Supplement: Additional file 5: Figure S3. — A comparison between the shared GOs detected by CSA:Drought and three independent Arabidopsis studies. [file 12870_2015_493_MOESM5_ESM.pdf]

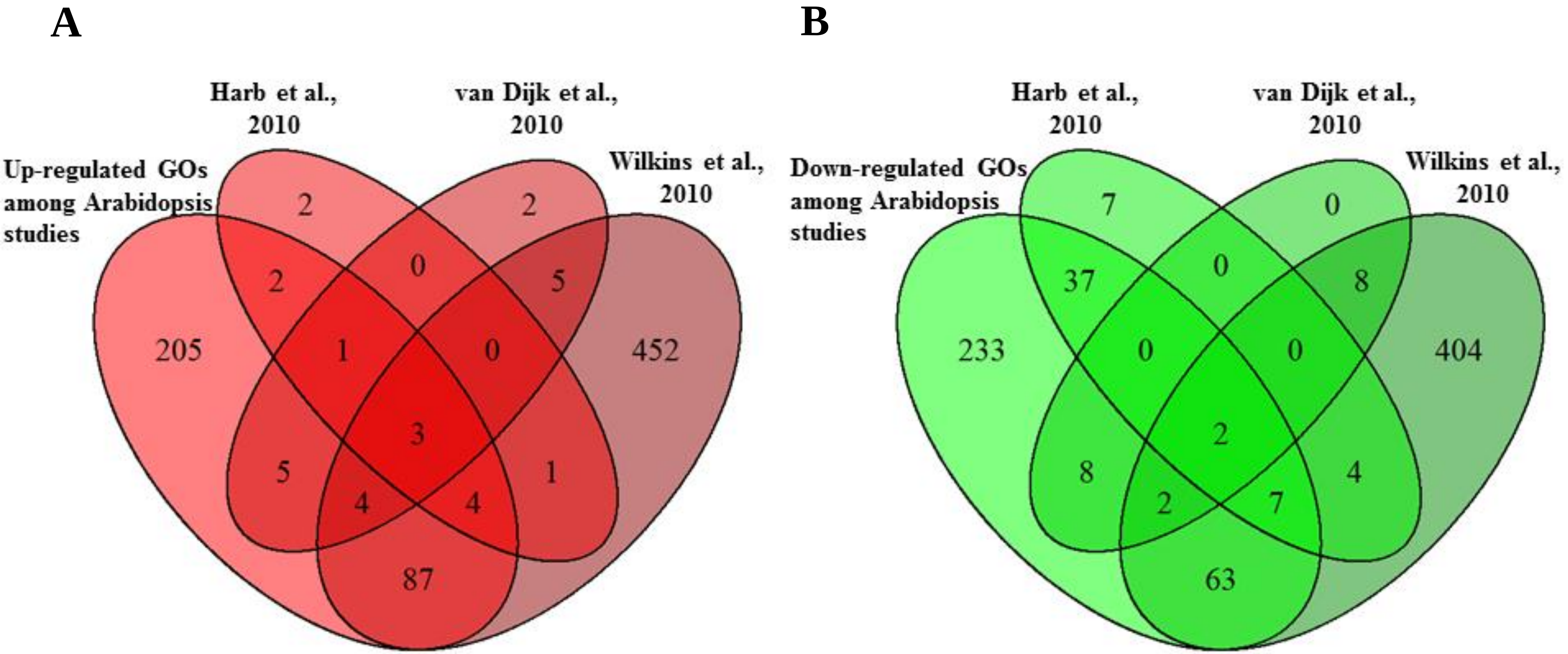

Additional file 5: Figure S3. Common and unique (**A**) up- and (**B**) down- regulated GOs among the shared GOs detected by CSA:Drought and by three independent GO lists, obtained from Arabidopsis studies included in the meta-analysis. GOs in each intersection are shown as numbers within Venn diagram circles.
