# Supplementary material for: Identification of conserved drought-adaptive genes using a cross-species meta-analysis approach
Source: BMC Plant Biol. 2015 May 3;15:111. doi: 10.1186/s12870-015-0493-6 (PMC4417316; doi:10.1186/s12870-015-0493-6)
Supplement: Additional file 9: Figure S4. — A comparison between the shared DEGs and independent lists obtained from (A) Arabidopsis or (B) wheat studies included in the meta-analysis. [file 12870_2015_493_MOESM9_ESM.pdf]

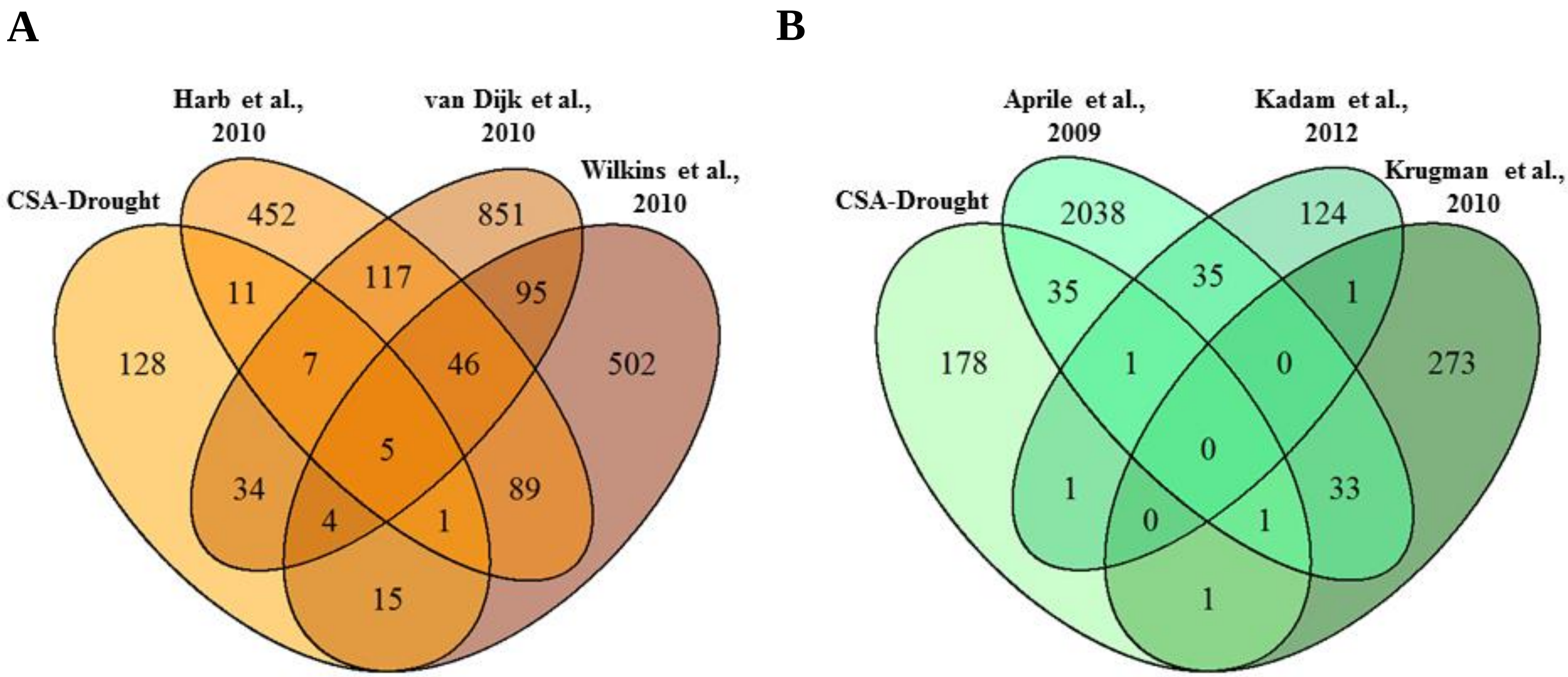

Additional file 9: Figure S4. A comparison between the shared drought adaptive DEGs and three independent drought DEG lists, retrieved from either (**A**) Wild type Arabidopsis or (**B**) wheat studies that were included in the meta-analysis. Common and unique differentially expressed genes are shown as numbers within Venn diagram circles.
