# Supplementary material for: Identification of conserved drought-adaptive genes using a cross-species meta-analysis approach
Source: BMC Plant Biol. 2015 May 3;15:111. doi: 10.1186/s12870-015-0493-6 (PMC4417316; doi:10.1186/s12870-015-0493-6)
Supplement: Additional file 12: Figure S5. — Enriched motifs in promoters of up-regulated shared drought-adaptive DEGs in (A) Arabidopsis and (B) rice. [file 12870_2015_493_MOESM12_ESM.pdf]

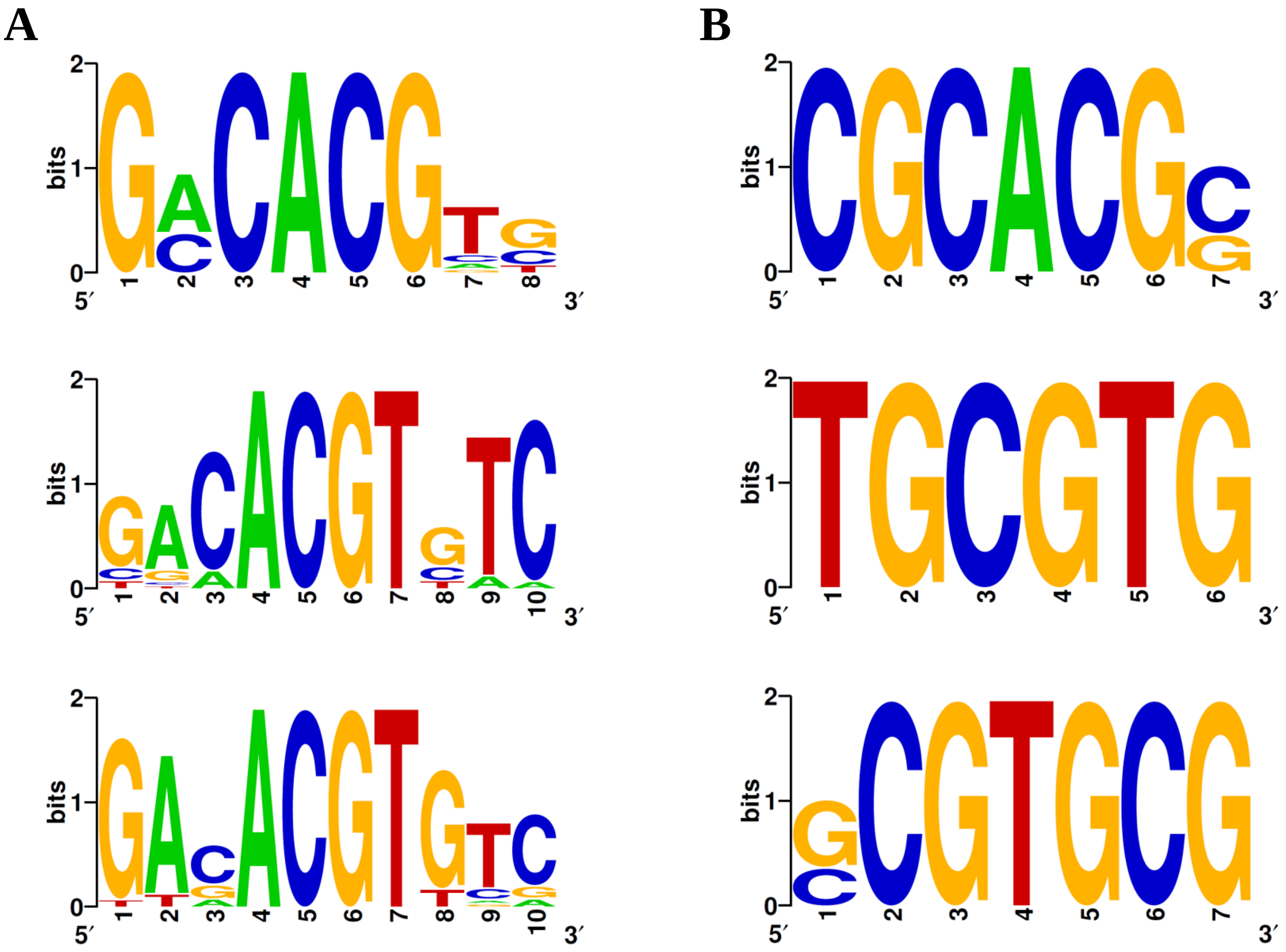

Additional file 12: Figure S5. Enriched motifs in promoters of up-regulated shared drought-adaptive DEGs in (**A**) Arabidopsis and (**B**) rice. The height of the letters is proportional to the frequency of the bases at each position. The sequence conservation is measured in bits, ranging from zero to maximum two bit score, and is reflected by the total height of each stack of letters.
