# Supplementary material for: Identification of conserved drought-adaptive genes using a cross-species meta-analysis approach
Source: BMC Plant Biol. 2015 May 3;15:111. doi: 10.1186/s12870-015-0493-6 (PMC4417316; doi:10.1186/s12870-015-0493-6)
Supplement: Additional file 13: Figure S6. — Relative expression of shared drought-adaptive orthologs under controlled and drought stressed Brachypodium distachyon plants. [file 12870_2015_493_MOESM13_ESM.pdf]

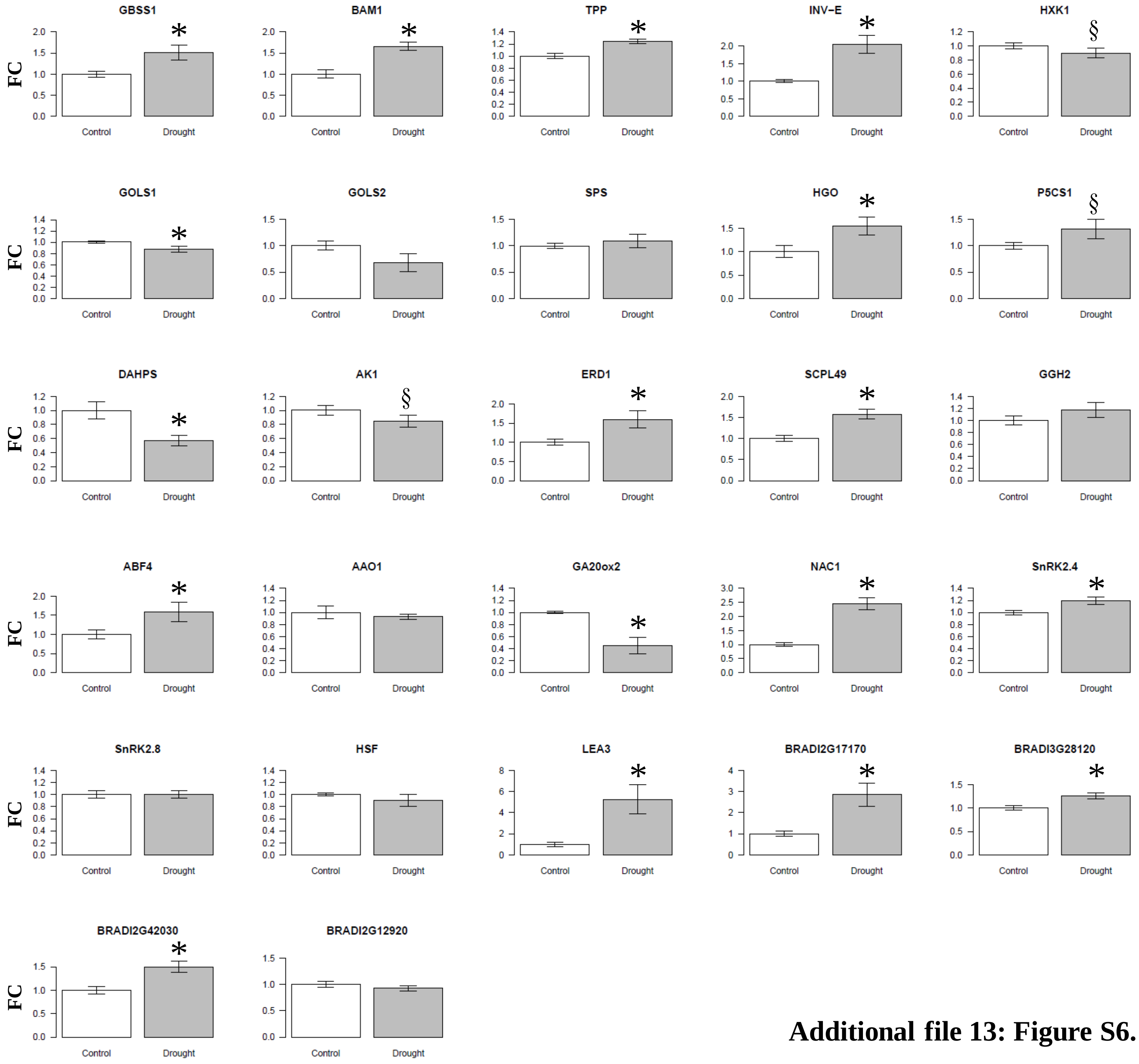

Additional file 13: Figure S6.

Additional file 13: Figure S6. Relative expression of shared drought-adaptive orthologs, representing known metabolic and regulatory pathways as well as unclassified genes, in controlled and drought-stressed *Brachypodium distachyon* plants. Mean values were calculated and normalized using two housekeeping genes *Glyceraldehyde 3-phosphate dehydrogenase* (*GAPDH*, BRADI3G14120) and *S-adenosylmethionine decarboxylase* (*SamDC*, BRADI5G14640) as internal controls and presented as fold-change. Values represent mean±SD (n=6). *GBSS1*, *Granule-bound starch synthase 1* (BRADI2G41590); *BAM1*, *β-Amylase 1* (BRADI1G75610); *TPP*, *Trehalose-6-phosphate phosphatase* (BRADI3G32970); *INV-E*, *Alkaline/neutral invertase E* (BRADI5G09200); *HXK1*, *Hexokinase 1* (BRADI2G18877); *GOLS1*, *Galactinol synthase 1* (BRADI1G64120); *GOLS2*, *Galactinol synthase 2* (BRADI1G17200); *SPS*, *Sucrose-phosphate synthase* (BRADI3G20120); *HGO*, *Homogentisate 1,2-dioxygenase* (BRADI1G52290); *P5CS1*, *Delta1-pyrroline-5-carboxylate synthetase* (BRADI2G23507); *DAHPS*, *3-Deoxy-D-arabino-heptulosonate 7-phosphate synthase* (BRADI1G21330); *AK1*, *Aspartate kinase 1* (BRADI1G01800); *ERD1*, *Early responsive to dehydration 1* (BRADI3G44640); *SCPL49*, *Serine carboxypeptidase-like 49* (BRADI3G01320); *GGH2*, *Gamma-glutamyl hydrolase 2* (BRADI2G19700); *ABF4*, *ABRE binding factor 4* (BRADI3G57960); *AAO1*, *Aldehyde oxidase 1* (BRADI1G06200); *SnRK2.4*, *SNF1-related kinase 2.4* (BRADI2G56267); *SnRK*2.8, *SNF1-related kinase 2.8* (BRADI1G60720); *GA20ox2*, *Gibberellin 20 oxidase 2* (BRADI1G14580); *NAC1*, *NAC domain containing protein 1* (BRADI4G02060); *HSF*, *Heat shock factor protein family* (BRADI1G38140); *LEA3*, *Late embryogenesis abundant protein, group 3* (BRADI2G18090). § and * indicate significant differences ($P \leq 0.1$ and $P \leq 0.05$) between treatments, respectively.
