# Supplementary material for: Identification of conserved drought-adaptive genes using a cross-species meta-analysis approach
Source: BMC Plant Biol. 2015 May 3;15:111. doi: 10.1186/s12870-015-0493-6 (PMC4417316; doi:10.1186/s12870-015-0493-6)
Supplement: Additional file 15: Figure S7. — Alteration in expression of carbohydrate and amino acid metabolic genes, involved in osmoregulation under drought, that were detected by CSA:Drought. [file 12870_2015_493_MOESM15_ESM.pdf]

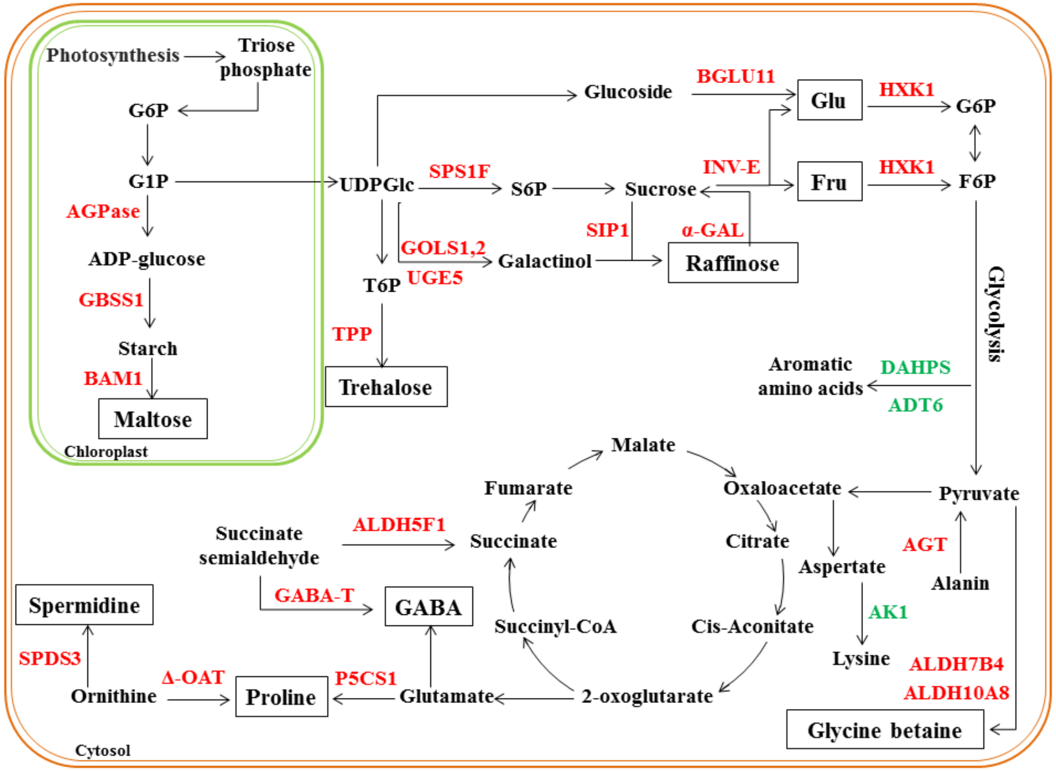

Additional file 15: Figure S7. Alteration in expression of carbohydrate and amino acid metabolic genes, involved in osmoregulation under drought. Designated in red and green are up- and down-regulated shared drought adaptive DEGs, respectively. Metabolites indicated with rectangles are known to be involved in osmoregulation. *AGPase, Glucose-1-phosphate adenylyltransferase*; *GBSS1, Granule-bound starch synthase 1*; *BAM1, β-Amylase 1*; *TPP, Trehalose-6-phosphate phosphatase*; *SPS1F, Sucrose phosphate synthase 1F, INV-E, Alkaline/neutral invertase E*; *BGLU11, β-Glucosidase 11*; *HXK1, Hexokinase 1, UGE5, UDP-D-galactose 4-epimerase 5*; *GOLS1,2, Galactinol synthase 1,2*; *SIP1, Seed imbibition 1*; *α-GAL, α-Galactosidase*; *DAHPS, 3-Deoxy-D-arabino-heptulosonate 7-phosphate synthase*; ADT6, *Arogenate dehydratase 6*; *AGT, Alanine-glyoxylate aminotransferase*; *AK1, Aspartate kinase 1*; *ALDH7B4, Aldehyde dehydrogenase 7B4*; *ALDH10A8, Betaine-aldehyde dehydrogenase 10A8*; *P5CS1, Delta1-pyrroline-5-carboxylate synthetase*; *Δ-OAT, Ornithine-oxo-acid transaminase*; *SPDS3, Spermidine synthase 3*; *GABA-T, 4-Aminobutyrate transaminase*; *ALDH5F1, Succinate-semialdehyde dehydrogenase*. G6P, Glucose 6-phosphate; G1P, Glucose 1-phosphate; UDPGlc, UDP-glucose; T6P, Trehalose 6-phosphate; S6P, Sucrose 6-phosphate; Glu, Glucose; Fru, Fructose; F6P, Fructose 1-phosphate.
